# Shared genetic architecture between irritable bowel syndrome and psychiatric disorders reveals molecular pathways of the gut-brain axis

**DOI:** 10.1186/s13073-023-01212-4

**Published:** 2023-08-01

**Authors:** Markos Tesfaye, Piotr Jaholkowski, Guy F. L. Hindley, Alexey A. Shadrin, Zillur Rahman, Shahram Bahrami, Aihua Lin, Børge Holen, Nadine Parker, Weiqiu Cheng, Linn Rødevand, Oleksandr Frei, Srdjan Djurovic, Anders M. Dale, Olav B. Smeland, Kevin S. O’Connell, Ole A. Andreassen

**Affiliations:** 1 NORMENT, Centre for Mental Disorders Research, Division of Mental Health and Addiction, Oslo University Hospital, Institute of Clinical Medicine, University of Oslo, Oslo, Norway; 2 NORMENT, Department of Clinical Sciences, University of Bergen, Bergen, Norway; 3 Institute of Psychiatry, Psychology and Neuroscience, King’s College London, London, UK; 4 KG Jebsen Centre for Neurodevelopmental Disorders, University of Oslo and Oslo University Hospital, Oslo, Norway; 5 Center for Bioinformatics, Department of Informatics, University of Oslo, Oslo, Norway; 6 Department of Medical Genetics, Oslo University Hospital, Oslo, Norway; 7 Department of Radiology, University of California, San Diego, La Jolla, CA USA; 8 Multimodal Imaging Laboratory, University of California San Diego, La Jolla, CA USA; 9 Department of Psychiatry, University of California, San Diego, La Jolla, CA USA; 10 Department of Neurosciences, University of California San Diego, La Jolla, CA USA

**Keywords:** Irritable bowel syndrome, Genetic overlap, Psychiatric disorder, Gut-brain axis

## Abstract

**Background:**

Irritable bowel syndrome (IBS) often co-occurs with psychiatric and gastrointestinal disorders. A recent genome-wide association study (GWAS) identified several genetic risk variants for IBS. However, most of the heritability remains unidentified, and the genetic overlap with psychiatric and somatic disorders is not quantified beyond genome-wide genetic correlations. Here, we characterize the genetic architecture of IBS, further, investigate its genetic overlap with psychiatric and gastrointestinal phenotypes, and identify novel genomic risk loci.

**Methods:**

Using GWAS summary statistics of IBS (53,400 cases and 433,201 controls), and psychiatric and gastrointestinal phenotypes, we performed bivariate casual mixture model analysis to characterize the genetic architecture and genetic overlap between these phenotypes. We leveraged identified genetic overlap to boost the discovery of genomic loci associated with IBS, and to identify specific shared loci associated with both IBS and psychiatric and gastrointestinal phenotypes, using the conditional/conjunctional false discovery rate (condFDR/conjFDR) framework. We used functional mapping and gene annotation (FUMA) for functional analyses.

**Results:**

IBS was highly polygenic with 12k trait-influencing variants. We found extensive polygenic overlap between IBS and psychiatric disorders and to a lesser extent with gastrointestinal diseases. We identified 132 independent IBS-associated loci (condFDR < 0.05) by conditioning on psychiatric disorders (*n* = 127) and gastrointestinal diseases (*n* = 24). Using conjFDR, 70 unique loci were shared between IBS and psychiatric disorders. Functional analyses of shared loci revealed enrichment for biological pathways of the nervous and immune systems. Genetic correlations and shared loci between psychiatric disorders and IBS subtypes were different.

**Conclusions:**

We found extensive polygenic overlap of IBS and psychiatric and gastrointestinal phenotypes beyond what was revealed with genetic correlations. Leveraging the overlap, we discovered genetic loci associated with IBS which implicate a wide range of biological pathways beyond the gut-brain axis. Genetic differences may underlie the clinical subtype of IBS. These results increase our understanding of the pathophysiology of IBS which may form the basis for the development of individualized interventions.

**Supplementary Information:**

The online version contains supplementary material available at 10.1186/s13073-023-01212-4.

## Background

Irritable bowel syndrome (IBS) is an enduring disorder of the intestine characterized by recurrent abdominal pain and bowel habit changes without identifiable pathology on clinical or laboratory examination [1, 2]. It is a common condition with a prevalence of 9.2% and is associated with significant morbidity and poor quality of life [3]. Several factors are implicated in the development of symptoms of IBS, including dysregulation of the gut-brain axis, disruption in intestinal permeability, dysbiosis, dysfunction of gut motility, and genetic and psychosocial factors [4, 5]. The knowledge gap in the pathophysiology of IBS hinders the development of effective treatments [6]. To this end, there is a need to advance genetic discoveries in IBS to improve our understanding of the pathophysiology of IBS at a molecular level [6].

Causal genetic factors are supported by an estimated heritability of 19.5 (± 8.5)% and an increased risk of IBS among biological children of individuals with IBS compared to adoptees [7]. However, the identification of genomic risk loci for complex traits such as IBS is limited by their polygenic architecture, which requires very large sample sizes to detect genetic variants with small effect sizes [8]. To date, genome-wide association studies (GWAS) of IBS identified a small number of genomic loci [9–11], leaving a large portion of the heritability due to common genetic variants undiscovered [12, 13]. Identification of enough common variants to explain a significant part of heritability is crucial for more meaningful application of discoveries [13] and for characterizing associated molecular pathways.

While larger sample sizes will increase genomic discoveries in highly polygenic traits [12, 14], such an undertaking requires investment of time and funding. Alternatively, advanced methods in statistical genetics have demonstrated the potential to boost the power of GWAS to increase the discovery of genomic risk loci by leveraging auxiliary genetic data to identify SNPs associated with a trait that did not initially reach the genome-wide significance threshold [15–17]. These methods take advantage of genetic overlap between two traits and have been successfully applied to improve genetic discovery across psychiatric and somatic phenotypes, such as cardiometabolic traits and major depression (MD) [18], schizophrenia (SCZ) and somatic traits [19], and bipolar disorder (BIP) and cardiovascular diseases [17, 20]. This boost in genetic discovery relies on the extent of genetic overlap between the pair of traits [17, 21].

The existing clinical and epidemiological data support comorbidity between IBS, and both psychiatric [22–24] and gastrointestinal diseases [25, 26]. We confirm and leverage these overlaps to discover novel genomic loci [17] for IBS, and thereby, advance the knowledge of the molecular pathways involved which can form the basis for development of new treatments [6]. Furthermore, comprehensive characterization of the genetic landscape of IBS and genetic overlap with other phenotypes can inform diagnostic nosology [27]. The bivariate causal mixture model (MiXeR) provides an estimate of the total number of unique and shared genetic variants for a pair of traits and quantifies the proportion of concordant variants within the shared component [21, 27].

Here, we applied advanced statistical methods to characterize the shared polygenic architecture of IBS and comorbid psychiatric and gastrointestinal phenotypes and leveraged this overlap to boost the power to identify more IBS loci [15]. First, we performed MiXeR analyses to elucidate the polygenic architecture of IBS and quantify the genetic overlap with clinically related psychiatric and gastrointestinal disorders. Second, we applied conditional FDR (condFDR) to boost the discovery of specific genetic loci or variants associated with IBS [15, 17], and conjunctional FDR (conjFDR) to identify shared loci using GWAS summary data. We hypothesized that the known clinical comorbidity between IBS and psychiatric and gastrointestinal disorders is in part related to shared genetic architecture. Hence, the genetic overlap can be leveraged to identify novel IBS-associated loci and reveal molecular pathways involved in the pathophysiology of IBS.

## Methods

### Genome-wide association studies (GWAS) data

#### Datasets for IBS﻿

The GWAS summary statistics for IBS were obtained from participants of the UK Biobank and the Belly Genes Initiative (BGI) who are of European Ancestry. The UK Biobank cases of IBS 40,548 were individuals who fulfilled the Rome III criteria of IBS on the Digestive Health Questionnaire (DHQ) (*n* = 24,845), reported to have received a diagnosis of IBS previously, or a diagnosis of ICD-10 IBS in their electronic medical records. The controls comprised 72,356 DHQ respondents and 220,864 DHQ non-respondents [11]. The BGI is an international collaboration of multiple cohorts of IBS with 12,852 cases and 139,981 controls. The diagnosis of IBS in the BGI sample was based on electronic medical records, specialist diagnoses from tertiary clinics, and questionnaire data (including Rome III criteria). Individuals with chronic intestinal diseases such as coeliac disease, and Crohn’s disease have been excluded from both case and control samples.

The IBS subtype-specific summary statistics comprised the DHQ respondents of the UK Biobank sample described above. Accordingly, the controls were 72,356 for all IBS subtypes, and the cases were: constipation-predominant (IBSC; *n* = 5406), diarrhea-predominant (IBSD; *n* = 8756), and with mixed constipation and diarrhea (IBSM; *n* = 17,216) [11].

#### Dataset for generalized anxiety disorder (GAD)

The GWAS summary statistics for GAD were obtained from the Million Veteran Program (MVP) cohort. A detailed description of the MVP cohort characteristics is available elsewhere [28]. In the GWAS of GAD, the phenotype was assessed using a dimensional self-report survey using the GAD-2 scale in 175,163 adults of European Ancestry [29].

#### Dataset for MD

The GWAS summary statistics for MD was obtained from a meta-analysis of three large GWAS of depression among populations of European Ancestry [30]. The meta-analyses comprised the GWAS of depression diagnosed using structural clinical interviews or similar criteria from the Psychiatric Genomics Consortium (PGC) (43,204 cases and 95,680 controls) [31], the GWAS of self-reported history of diagnosis of depression from 23andMe, Inc. (75,607 cases and 231,747 controls) [32], and the GWAS of a broad depression phenotype from the UK Biobank (127,552 cases and 233,763 controls) [33].

#### Dataset for BIP

The GWAS summary statistics for BIP was obtained from the third wave of the PGC comprising 57 cohorts collected in Europe, North America, and Australia [34]. The total sample was 41,917 cases and 371,549 controls of European Ancestry. Cases were defined as individuals meeting one of the international consensus criteria (DSM-IV, ICD-9, or ICD-10) for a lifetime diagnosis of BIP using structured diagnostic instruments. Some cohorts obtained from biobanks had the BIP cases ascertained using ICD codes or self-report.

#### Dataset for SCZ

The GWAS summary statistics for SCZ comprised the European subset of the PGC meta-analysis of cohorts of schizophrenia and schizoaffective disorder. The sample used for this GWAS includes 53,386 cases and 77,258 controls [35].

#### Datasets for diverticular disease (DVD)

Two GWAS summary statistics of DVD in populations of European Ancestry were used. The dataset from the sixth version of the Finnish national biobank (FinnGen) had 17,851 cases of diverticular disease of the intestine based on ICD-9 or ICD-10 of hospital records, and 14,357 controls [36]. The second dataset was from the European sample of the UK biobank with 27,444 cases based on the ICD codes and 382,284 controls [37].

#### Datasets for inflammatory bowel disease (IBD)

The GWAS summary statistics of IBD were obtained from a meta-analysis reported by the international IBD genomics consortium on a population of European Ancestry. The meta-analysis comprised 25,042 clinically ascertained cases (12,194 Crohn’s disease and 12,366 ulcerative colitis), and 34,915 controls [38].

In all GWAS datasets except that of IBS, we excluded samples from the UK Biobank from phenotypes other than IBS to avoid potential sample overlap as required by polygenic enrichment analyses (Table 1). Since MiXeR accounts for sample overlap, we used the whole samples without removal of overlapping samples [21].

**Table 1:** List of genome-wide association study data used

Trait/phenotype	Analysis	Number of cases	Number of controls	Ancestry of participants	Source of data
**Psychiatric**					
SCZ	MiXeR and cFDR	53,386	77,258	European	PGC-SCZ [35]
BIP	MiXeR	41,917	371,549	European	PGC-BIP + UKBB [34]
	cFDR	40,463	313,436	European	PGC-BIP [34] PGC-MD + 23andMe + UKBB [30]
MD	MiXeR	246,363	561,190	European	
	cFDR	121,198	329,421	European	PGC-MD + 23andMe [31]
GAD	MiXeR and cFDR	175,163	Not applicable	European	MVP [29]
IBS	MiXeR and cFDR	53,400	433,201	European	UKBB + BGI [11]
IBS-C	cFDR	5406	72,356	European	UKBB [11]
IBS-D	cFDR	8756	72,356	European	UKBB [11]
IBS-M	cFDR	17,216	72,356	European	UKBB [11]
**Somatic**					
IBD	MiXeR & cFDR	25,042	34,915	European	IIBDGC-IBD [38]
DVD	cFDR	17,851	14,357	European	FinnGen [36]
	MiXeR	27,444	382,284	European	UKBB [37]
Height	MiXeR	709,706	Not applicable	European	GIANT + UKBB [39]

*MiXeR* bivariate causal mixture analyses, *cFDR* conditional false discovery rate analyses, *PGC* Psychiatric Genomics Consortium, *SCZ* schizophrenia, *BIP* bipolar disorder, *MD* major depression, *GAD* generalized anxiety disorder, *IBS* irritable bowel syndrome, *IBS-C* constipation-predominant IBS, *IBS-D* diarrhea-predominant IBS, *IBS-M* IBS with mixed constipation and diarrhea *MVP* Million Veterans Program, *UKBB* UK Biobank, *BGI* Belly Genes Initiative, *IIBDGC* International Inflammatory Bowel Disease Genetics Consortium, *IBD* inflammatory bowel disease, *DVD* diverticular disease, FinnGen Finnish biobank project, *GIANT* Genetic Investigation of Anthropometric Traits Consortium

### Statistical analysis

We constructed quantile–quantile (Q-Q) plots by conditioning IBS on each of the phenotypes and inspected the plots for polygenic enrichment. We applied casual mixture models to investigate the genetic overlap between IBS, and each of GAD, MD, BIP, SCZ, DVD, and IBD using MiXeR [21]. We carried out univariate MiXeR analyses to estimate the number of trait-influencing variants (i.e., variants with genetic effects which are not due to linkage disequilibrium (LD)). Univariate MiXeR model assumes that common genetic variants can be causal or non-causal to the specific trait. Hence, the polygenicity, the number of “causal” variants that explain 90% of SNP heritability, is computed using maximum likelihood estimation. Bivariate MiXeR builds on the univariate model for a pair of traits and thereby estimates of trait-specific and shared “causal” variants are estimated. Unlike genetic correlations, the estimates of shared ‘causal’ variants are independent of the effect directions on the pair of traits [21]. MiXeR also estimates dice coefficient scores (i.e., the proportion of shared variants between two traits out of the total number of variants estimated to influence both traits) and computes the fraction of variants with concordant effects among the shared component (Additional file 1). We performed MiXeR analysis for IBS and height as a heritable somatic comparator. We computed the genetic correlations of IBS and the other phenotypes using LD score regression [40].

We utilized the condFDR method to identify loci associated with IBS by conditioning genetic associations with IBS on each of the psychiatric and gastrointestinal phenotypes [15–17]. The condFDR method is an extension of the standard FDR and builds on an empirical Bayesian statistical framework to exploit the power of combining two GWASs for improving the discovery of genetic variants [17]. The method leverages the presence of SNP associations with the primary and conditional phenotypes [41] to identify variants more likely to be true associations even though the p-values do not reach the genome-wide significance threshold [15, 17]. CondFDR procedure re-ranks the SNP p-values in a primary phenotype (IBS) based on their associations in a conditional phenotype (e.g., SCZ). Hence, IBS variants jointly associated with SCZ will obtain lower condFDR estimates [15–17]. Similarly, conjFDR enables the detection of SNPs associated with both the primary and conditional phenotypes, based on inverse condFDR analyses in which the primary phenotype became conditional, and the conditional phenotype became the primary phenotype. The conjFDR statistic is defined as the maximum of two condFDR values (e.g., IBS conditional on SCZ and vice versa) [17]. For our analyses, we used an FDR threshold of 5% for condFDR and conjFDR. Due to complex LD which can potentially bias FDR estimation [42], we excluded SNPs within the extended major histocompatibility complex region and chromosome 8p23.1 (genome build 19 positions of chr6:25119106–33854733 and chr8:7200000–12500000, respectively) before fitting the FDR models. We randomly pruned SNPs in 500 iterations to minimize inflation in fold enrichment. Hence, one candidate SNP was randomly selected for an LD block (*r*^2^ > 0.1) and the respective p-values were used to compute the empirical cumulative distribution functions (Additional file 1).

### Definition of genomic loci

We defined independent genomic loci based on the functional mapping and gene annotation (FUMA) protocol [43]. We considered candidate SNPs with condFDR/conjFDR < 0.05 and LD *r*^2^ < 0.6 with each other as independent significant SNPs, and we designated those with LD *r*^2^ < 0.1 as lead SNPs. All the candidate SNPs in LD *r*^2^ ≥ 0.6 with a lead SNP demarcated the boundaries of a genomic locus. We merged loci separated by less than 250 kb. Thus, we defined any candidate SNP located within the boundaries of a genomic locus to belong to a single independent genomic locus. We computed LD information from the 1000 Genomes Project reference panel [44]. For the shared loci, we inferred the effect directions by comparing the *z*-scores in the GWAS summary statistics corresponding to the phenotype. We considered loci not identified in the GWAS catalog (downloaded in April 2022) and in previous IBS studies as novel risk loci [11, 45].

### Replication of condFDR significant loci in independent samples

Due to the small genetic effects of individual lead SNPs and the consequent low probability of replicating specific genome-wide significant loci in a smaller replication dataset, we tested for sign concordance of effect direction in the primary IBS GWAS dataset and a replication GWAS of IBS from FinnGen [36]. Previous studies have utilized a similar approach [35, 46, 47]. If lead SNPs were missing in the replication GWAS data set, we replaced them with the next most significant candidate SNP, if available. We then tested the significance of whether the sign concordance was randomly distributed using a one-sided exact binomial test.

### Functional annotations

We carried out positional annotation for all lead SNPs with a condFDR/conjFDR < 0.05 using FUMA [43]. SNPs were annotated for Combined Annotation Dependent Depletion (CADD) scores to estimate the deleteriousness of the SNP on protein function [48], and RegulomeDB scores provided predicted regulatory functionality of the SNP [49]. Functional gene mapping was performed for all lead SNPs from condFDR/conjFDR using the OpenTargets platform [50]. For each SNP, we used the one gene with the highest score on the OpenTargets mapping procedure for gene ontology (GO) analyses. GO for IBS was analyzed including all genes mapped to each lead SNP identified on condFDR analyses. Also, GO was performed for genes from the functional mapping of shared lead SNPs from conjFDR of IBS and psychiatric disorders. Both the GO analyses were performed using FUMA [43]. The shared loci between IBS and somatic phenotypes were too few for GO analyses.

## Results

### Cross-phenotype polygenic enrichment

**Fig. 1:**
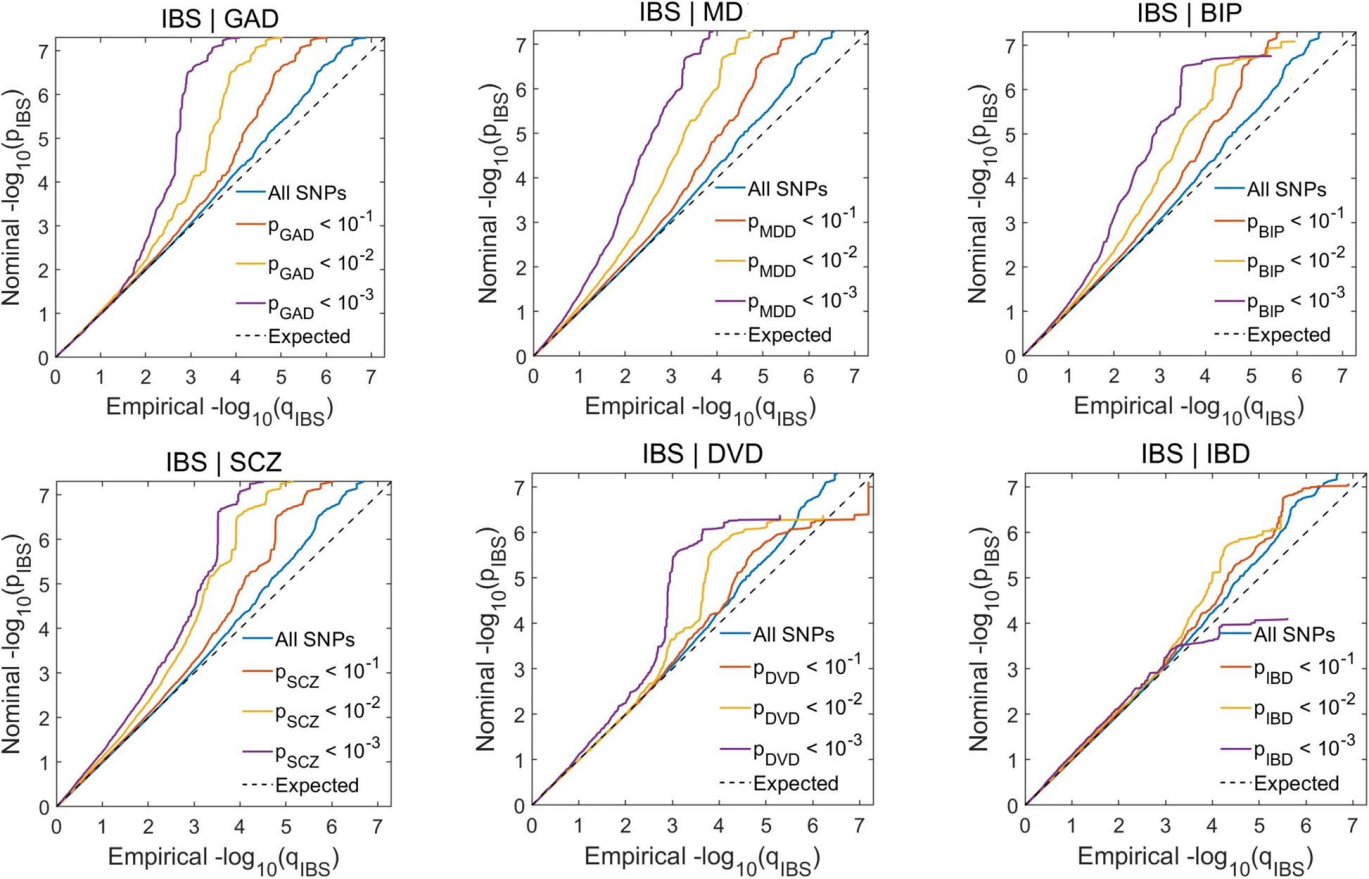
Conditional Q-Q plots of nominal –log10 *p*-values vs empirical –log10 *p*-values in irritable bowel syndrome (IBS) below the standard genome-wide association study threshold of *p* < 5.0 × 10^−8^ as a function of significance of association with generalized anxiety disorder (GAD), major depression (MD), bipolar disorder (BIP), schizophrenia (SCZ), diverticular disease (DVD), or inflammatory bowel disease (IBD) below the level of –log10 *p*-values of 1, 2, or 3, corresponding to *p* < 0.10, *p* < 0.01 and *p* < 0.001, respectively. The blue line includes all SNPs and dashed lines indicate the null hypothesis

We examined the Q-Q plots for cross-trait polygenic enrichment which manifests as an upward and leftward deflection of the plots for subsets of SNPs increasingly associated with the secondary phenotype [17]. We found that the Q-Q plots of *p*-values from IBS conditioned on the *p*-value strata from MD, BIP, SCZ, and GAD GWAS data exhibited polygenic enrichment. However, there was less polygenic enrichment on Q-Q plots of SNP nominal *p*-values from IBS when stratified based on the *p*-values of DVD or IBD GWAS data compared to those conditioned on psychiatric disorders (Fig. 1). The inverse Q-Q plots with IBS as secondary phenotype also showed similar patterns enrichment (Additional file 2: Fig. S1).

### Polygenicity, genetic overlap, and correlation

**Fig. 2:**
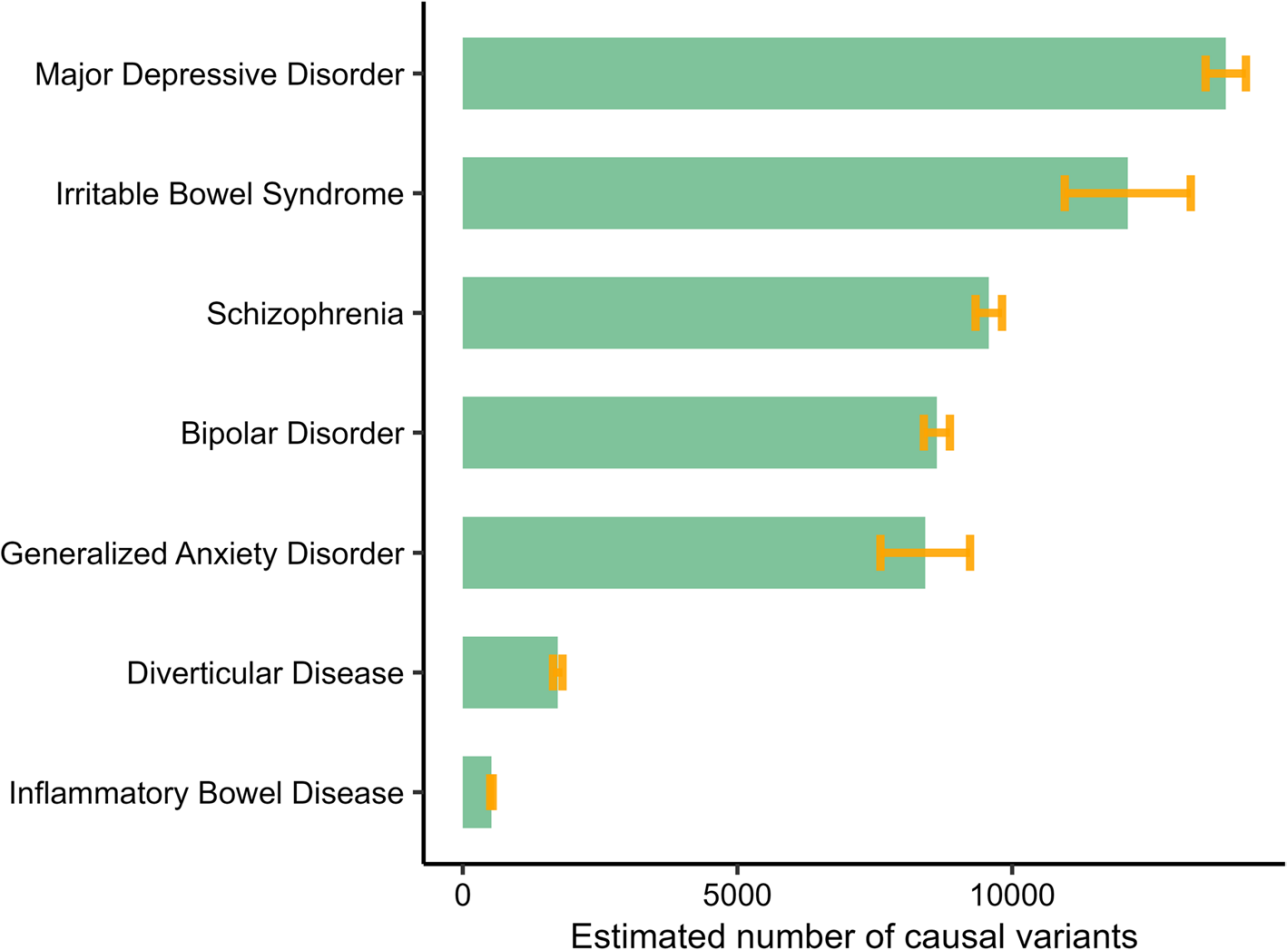
Polygenicity of irritable bowel syndrome (IBS), generalized anxiety disorder (GAD), major depression (MD) bipolar disorder (BIP), schizophrenia (SCZ), diverticular disease (DVD), inflammatory bowel disease (IBD), and height (HGT) with the number of trait-influencing (“causal”) variants explaining 90% of the heritability (estimated from univariate MiXeR analysis)

In univariate MiXeR analysis, we estimate that IBS is a highly polygenic phenotype with approximately 12.1 k ± 1.1 k “trait-influencing” variants which account for 90% of heritability. The corresponding values for the psychiatric phenotypes were GAD (8.4 k ± 0.8 k), MD (13.9 k ± 0.4 k), BIP (8.6 k ± 0.2 k) and SCZ (9.6 k ± 0.2 k). The somatic phenotypes were less polygenic with the number of trait-influencing variants for IBD (0.5 k ± 22) and DVD (1.7 k ± 86) (Fig. 2). The SNP heritability of IBS was approximately 0.06 (SD = 0.001), which is similar to that of MD (Additional file 3: Tables S1 – S8). The GWAS of IBS subtypes did not have adequate power for MiXeR analysis.

**Fig. 3:**
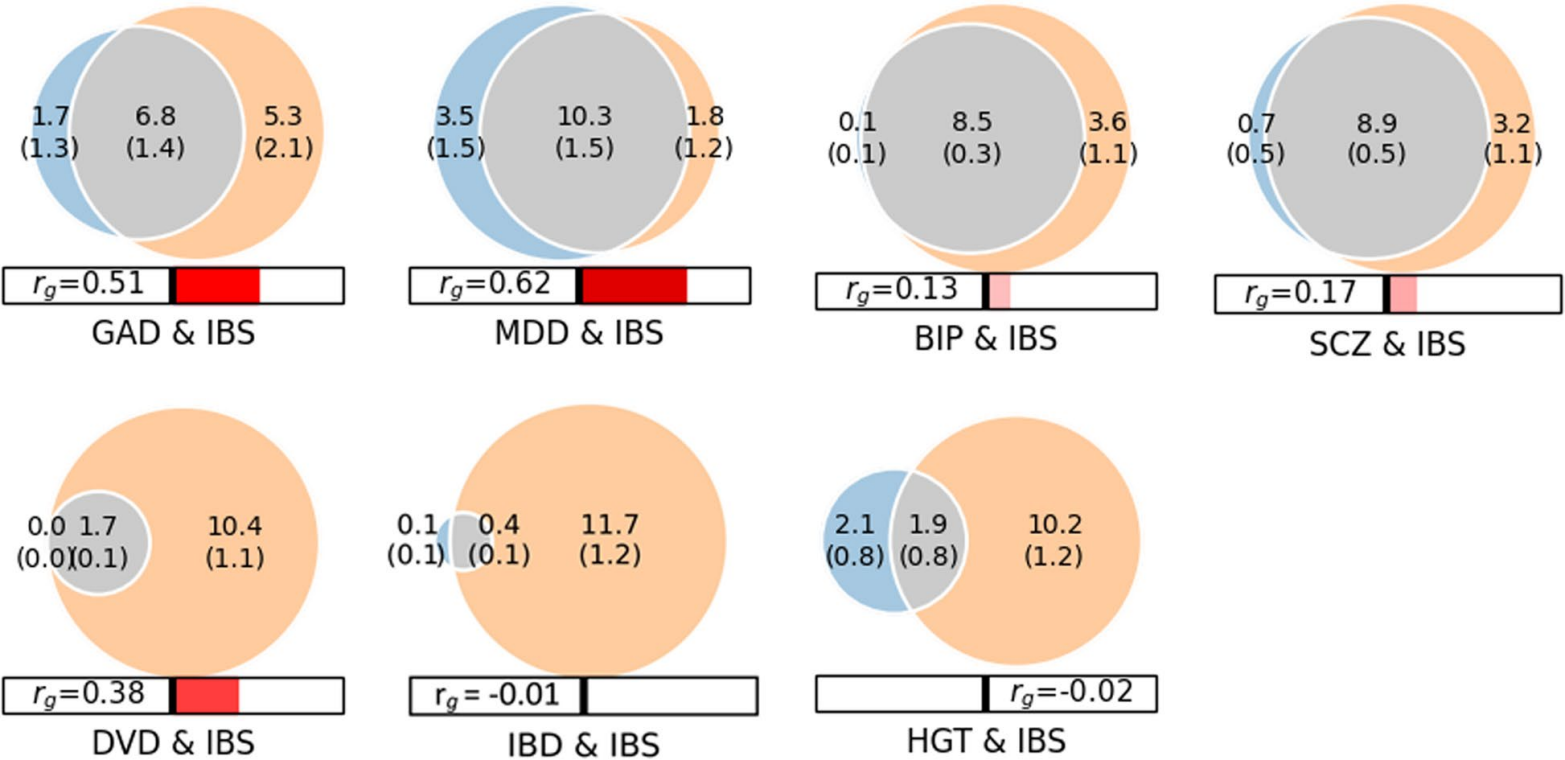
Genome-wide genetic overlap and genetic correlation among irritable bowel syndrome (IBS), generalized anxiety disorder (GAD), major depression (MD) bipolar disorder (BIP), schizophrenia (SCZ), diverticular disease (DVD), inflammatory bowel disease (IBD), and Height (HGT). The numbers in colored Venn diagrams indicate the number of shared and phenotype-specific trait-influencing variants which account for 90% of heritability in thousands, and r_g_ represents genome-wide genetic correlation. N.B. For GAD, MD, and DVD, the value of minimum AIC was negative — an indicator that the shared component may be smaller than shown in the figure model fit (MiXeR analysis)

Bivariate MiXeR revealed extensive genetic overlap between IBS and psychiatric disorders (Fig. 3). The estimated number of shared trait-influencing variants between IBS and BIP was 8.5 k ± 0.3 k with 55% of them having concordant effects. The corresponding estimates for IBS and SCZ were 8.9 k ± 0.5 k variants with 56% having concordant effects. IBS and GAD had an estimated 6.8 k ± 1.4 k shared trait-influencing variants and 81% of these had concordant effects. Also, IBS and MD had an estimated 10.3 k ± 1.5 k shared trait-influencing variants of which 80% had concordant effects (Additional file 3: Tables S9 – S15). Notably, we found fewer shared trait-influencing variants between IBS and the somatic phenotypes; an estimated 0.4 k ± 0.1 k with IBD and 52% having the same effect directions, and approximately 1.7 k ± 86 with DVD and almost all (99%) having concordant effects (Fig. 3).

The degree of genetic overlap as estimated by the dice coefficient between IBS and psychiatric phenotypes were GAD (66%), MD (79%), BIP (82%), and SCZ (83%). The genetic overlap (dice coefficient) between IBS and somatic phenotypes was DVD (25%) and IBD (6%), while the corresponding figure between IBS and the comparator phenotype height was 24% (Fig. 3). To account for the large differences in the polygenicity of secondary phenotypes, we computed the proportion of observed overlap in trait-influencing variants relative to the maximum possible overlap by dividing the number of variants in the shared component by the number of variants in the less polygenic of the two phenotypes in consideration. Consequently, the proportion of overlap for IBS and psychiatric phenotypes was GAD (80%), MD (85%), BIP (98%), and SCZ (93%). The proportions for IBS and the somatic phenotypes were DVD (100%), IBD (77%), and height (47%). The MiXeR results between IBS and MD, GAD and DVD are suboptimal model fit with negative Akaike information criterion (AIC) scores when comparing the best fitting model to the least possible overlap (minimum), indicating that the shared component between IBS and these traits may be smaller than what is estimated.

**Fig. 4:**
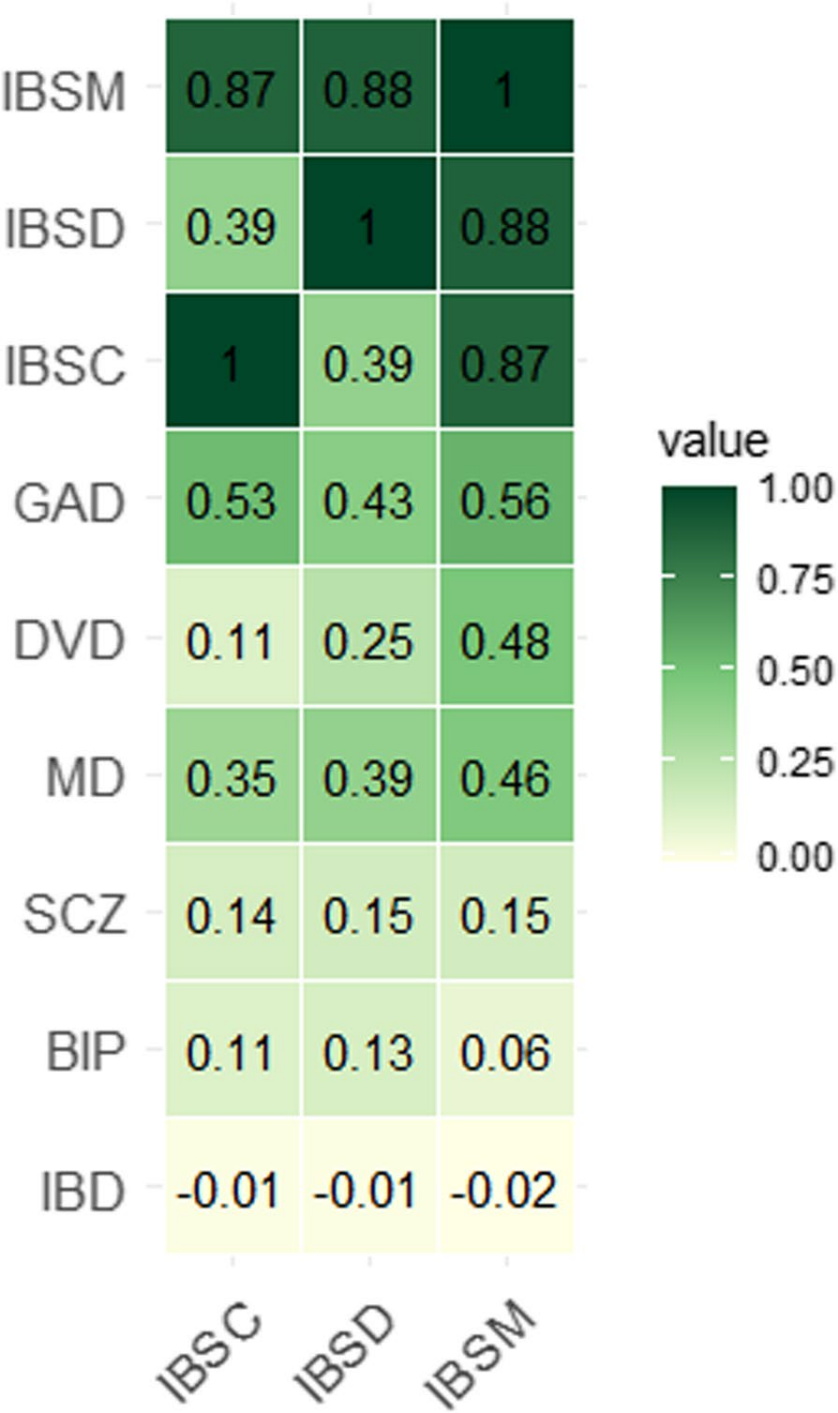
Genetic correlations from LD score regression analyses for subtypes of irritable bowel syndrome (IBS): IBS with constipation (IBSC), IBS with diarrhea (IBSD) and IBS with mixed constipation and diarrhea (IBSM), and psychiatric and gastrointestinal diseases. Generalized anxiety disorder (GAD), major depression (MD), bipolar disorder (BIP), schizophrenia (SCZ), diverticular disease (DVD), or inflammatory bowel disease (IBD)

In the LD-score regression analysis, IBS showed significant (*P* < 0.0001) genetic correlations with GAD (*r*_*g*_ = 0.49), MD (*r*_*g*_ = 0.55), BIP (*r*_*g*_ = 0.13), SCZ (*r*_*g*_ = 0.17), and DVD (*r*_*g*_ = 0.38), but not for IBD (*r*_*g*_ = -0.01). All three subtypes of IBS showed positive genetic correlation with SCZ, MD, and GAD (*P* < 0.05). None of the IBS subtypes exhibited a significant genetic correlation with IBD. BIP had a positive genetic correlation only with IBSD, and DVD showed a genetic correlation with both IBSD and IBSM. Interestingly, IBSD and IBSC showed a similar high genetic correlation with IBSM (*r*_*g*_ = 0.88, *P* < 0.00001); however, the genetic correlation between IBSD and IBSC was only moderate (*r*_*g*_ = 0.39, *P* < 0.01) (Fig. 4; Additional file 4: Table S16).

### Identification of genetic loci for IBS

We exploited the cross-trait enrichment of SNPs associated with IBS and psychiatric disorders using condFDR. We identified a total of 127 genomic risk loci for IBS by conditioning on GAD, MD, BIP or SCZ (condFDR < 0.05), and 111 of these loci were novel for IBS. In these condFDR analyses, we identified 36 loci with GAD, 69 loci with MD, 53 loci with BIP, and 41 loci with SCZ. In condFDR analysis of IBS leveraging associations with DVD or IBD, we identified 24 genomic loci associated with IBS including 14 novel risk loci. Specifically, 17 loci were identified conditional on DVD, and 15 loci conditional on IBD. Five of the 14 novel loci were not identified in condFDR analyses leveraging SNP associations with the psychiatric disorders—resulting in a total of 116 novel loci identified for IBS (Additional file 2: Fig. S2; Additional file 5: Tables S17 – S22).

### Identification of shared genetic loci for IBS and psychiatric disorders

**Fig. 5:**
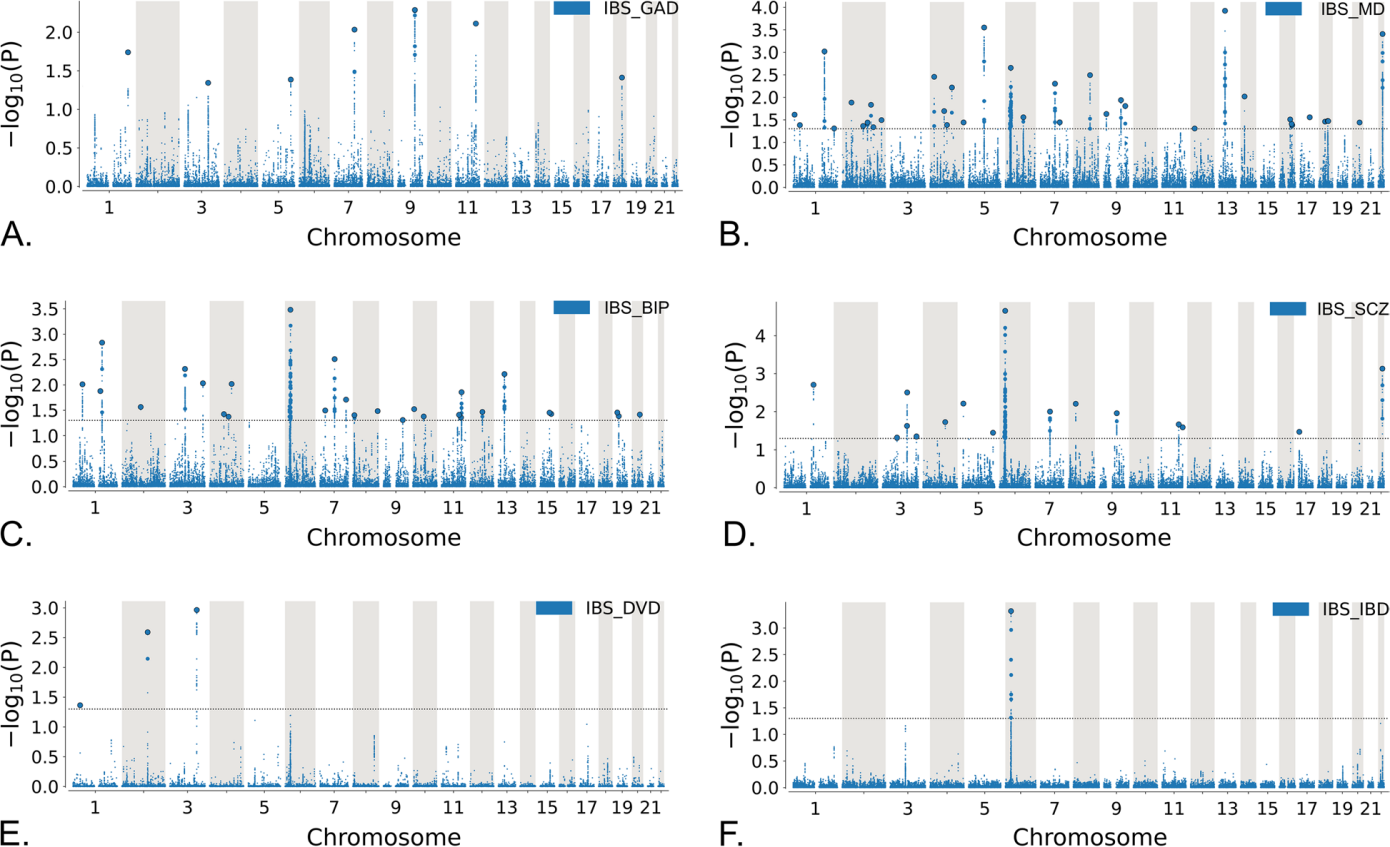
**A**–**F** Conjunctional FDR Manhattan plot of –log10 (conjFDR) values for loci shared between irritable bowel syndrome (IBS) and other phenotypes: generalized anxiety disorder (GAD), major depression (MD), bipolar disorder (BIP), schizophrenia (SCZ), diverticular disease (DVD) and inflammatory bowel disease (IBD) SNPs with conjunctional –log10(conjFDR) > 1.3 (i.e., conjFDR < 0.05) are shown with large points

Using conjFDR, we identified a total of 70 unique genomic loci shared between IBS and psychiatric disorders—seven with GAD, 35 with MD, 27 with BIP, and 15 with SCZ. All shared loci between IBS and GAD, and IBS and MD had concordant effect directions. Also, the majority of shared loci between IBS and BIP (20/27, 74.1%), and between IBS and SCZ (10/15, 66.7%) had concordant effect directions (Table 2; Fig. 5; Additional file 5: Tables S23—S26).

**Table 2:** ConjFDR analyses results of shared loci associated with irritable bowel syndrome (IBS), generalized anxiety disorder (GAD), major depression (MD), bipolar disorder (BIP), schizophrenia (SCZ), diverticular disease (DVD), and inflammatory bowel disease (IBD) (FDR < 0.05)

Locus	SNP	CHR	A1/A2	Mapped gene in OpenTargets	Function	ConjFDR IBS and GAD	ConjFDR IBS and MD	ConjFDR IBS and BIP	ConjFDR IBS and SCZ	ConjFDR IBS and DVD	ConjFDR IBS and IBD	*P*-value in IBS GWAS
1	rs5174	1	T/C	LRP8	Exonic	7.20E − 01	4.74E − 01	**9.73E − 03**	8.33E − 02	8.70E − 01	8.70E − 01	3.36E − 06
4^a^	rs4846898	1	A/G	GALNT2	Intronic	6.73E − 02	**4.91E − 02**	8.45E − 01	7.98E − 02	8.98E − 01	2.35E − 01	2.60E − 04
4^a^	rs552025051	1	T/TA	GALNT2	Intronic	**1.82E − 02**	NA	NA	NA	NA	NA	5.73E − 05
7^a^	rs2731562	2	A/C	ARHGAP15	Intronic	7.23E − 01	1.00E + 00	7.55E − 01	6.95E − 01	**2.56E − 03**	8.65E − 01	3.66E − 06
8^a^	rs197261	2	A/G	TANK	Downstream	4.10E − 01	**1.47E − 02**	8.08E − 02	8.56E − 01	9.32E − 01	9.17E − 01	1.05E − 05
11	rs9816652	3	A/G	CADM2	Intronic	1.00E + 00	8.84E − 01	**4.84E − 03**	7.18E − 01	9.63E − 01	8.34E − 01	8.61E − 06
12^a^	rs147538844	3	T/TC	PPP2R3A	Intergenic	**4.53E − 02**	NA	9.22E − 01	NA	7.92E − 01	NA	8.36E − 05
12^a^	rs9871437	3	A/G	PPP2R3A	Intergenic	8.17E − 02	8.04E − 01	1.00E + 00	**2.39E − 02**	9.01E − 01	8.64E − 01	4.05E − 05
14^a^	rs9855202	3	A/G	P2RY14	Intronic	1.00E + 00	9.79E − 01	8.04E − 01	9.67E − 01	**1.08E − 03**	8.50E − 01	1.46E − 06
15	rs10014468	4	A/G	IL21	Intergenic	3.79E − 01	**6.04E − 03**	**9.58E − 03**	2.83E − 02	1.00E + 00	8.47E − 01	2.21E − 05
15	rs45510091	4	A/G	KIAA1109	Intronic	2.26E − 01	7.41E − 03	1.17E − 02	**1.89E − 02**	9.98E − 01	8.29E − 01	2.97E − 05
16^a^	rs4362911	5	T/G	ADCY2	Intergenic	7.82E − 01	5.49E − 01	8.56E − 01	**6.16E − 03**	9.05E − 01	1.00E + 00	7.20E − 06
17^a^	rs4388243	5	A/G	PWWP2A	Intronic	**4.11E − 02**	5.75E − 01	1.00E + 00	7.76E − 01	9.10E − 01	1.00E + 00	9.29E − 05
20^a^	rs2140619	7	A/G	FOXP2	Intronic	**9.25E − 03**	9.04E − 01	9.71E − 01	9.19E − 01	9.13E − 01	1.00E + 00	1.57E − 05
21^a^	rs4726813	7	A/G	CNTNAP2	Intronic	7.78E − 01	3.35E − 01	**1.95E − 02**	6.58E − 01	NA	NA	4.53E − 05
22^a^	rs898797	8	T/C	MFHAS1	ncRNA_intronic	9.08E − 01	8.53E − 01	**3.95E − 02**	4.59E − 01	9.01E − 01	9.85E − 01	1.67E − 04
23^a^	rs13263876	8	A/G	TCIM	ncRNA_intronic	9.34E − 01	5.91E − 01	7.61E − 01	**6.24E − 03**	9.48E − 01	8.65E − 01	7.30E − 06
24^a^	rs4478545	8	A/G	PDP1	ncRNA_intronic	6.67E − 01	**3.21E − 03**	9.76E − 01	9.94E − 01	9.99E − 01	8.74E − 01	1.36E − 06
25	rs10821132	9	A/G	FAM120AOS	Intronic	2.61E − 01	1.38E − 01	3.80E − 01	**1.10E − 02**	9.01E − 01	8.67E − 01	3.02E − 07
25	rs10992790	9	T/C	FAM120AOS	Intronic	5.99E − 02	**1.15E − 02**	3.13E − 01	1.90E − 02	9.01E − 01	8.65E − 01	3.82E − 08
25	rs2265576	9	T/C	FAM120AOS	Intronic	**5.14E − 03**	8.12E − 01	1.00E + 00	9.52E − 01	NA	1.00E + 00	3.73E − 07
26^a^	rs60018597	9	T/C	TLR4	Intergenic	7.10E − 01	**1.56E − 02**	5.33E − 01	8.60E − 01	9.93E − 01	8.45E − 01	8.81E − 05
29	rs1836799	11	T/C	NCAM1	Intronic	2.04E − 01	7.22E − 01	**1.39E − 02**	2.29E − 02	9.01E − 01	9.96E − 01	3.84E − 05
29	rs2155285	11	A/G	NCAM1	Intronic	1.92E − 01	8.55E − 01	2.53E − 02	**2.18E − 02**	9.92E − 01	7.51E − 01	1.89E − 06
29	rs4937872	11	A/G	NCAM1	Intergenic	**7.72E − 03**	4.07E − 01	5.97E − 02	1.71E − 01	9.04E − 01	9.94E − 01	3.21E − 08
30	rs1373277	13	A/G	OLFM4	Intergenic	NA	2.04E − 02	**6.11E − 03**	9.85E − 01	9.01E − 01	8.12E − 01	1.18E − 05
30	rs1909650	13	A/G	OLFM4	Intergenic	7.62E − 01	**1.19E − 04**	1.18E − 01	6.62E − 01	9.01E − 01	8.01E − 01	1.53E − 07
32	rs67505447	14	A/G	LRFN5	Intronic	1.91E − 01	**9.58E − 03**	NA	NA	NA	1.00E + 00	4.29E − 05
33^a^	rs62083383	18	A/G	DCC	Intronic	**3.87E − 02**	7.98E − 01	9.19E − 01	1.00E + 00	9.10E − 01	9.53E − 01	1.57E − 04
34	rs4801153	18	T/C	TCF4	Intronic	2.70E − 01	**3.37E − 02**	1.00E + 00	NA	NA	7.43E − 01	2.86E − 04
36	rs20551	22	A/G	EP300	Exonic	7.18E − 01	**3.91E − 04**	3.24E − 01	**7.38E − 04**	9.67E − 01	5.93E − 01	6.16E − 07
37^a^	rs301805	1	T/G	RERE	Intronic	9.65E − 01	**2.43E − 02**	9.56E − 01	6.29E − 02	8.42E − 01	1.00E + 00	1.72E − 04
38^a^	rs11264103	1	A/G	INPP5B	Upstream	5.64E − 01	**4.14E − 02**	1.23E − 01	2.78E − 01	9.81E − 01	9.44E − 01	3.94E − 04
39	rs10917847	1	A/G	PBX1	Intergenic	8.42E − 01	1.47E − 01	**1.47E − 03**	**1.97E − 03**	1.00E + 00	8.76E − 01	1.88E − 06
40	rs2175177	1	A/G	COP1	ncRNA_intronic	9.80E − 01	**9.54E − 04**	1.82E − 01	8.20E − 01	8.37E − 01	9.83E − 01	1.84E − 06
42^a^	rs12713172	2	T/C	−	ncRNA_intronic	8.85E − 01	**1.31E − 02**	8.30E − 01	8.78E − 01	9.01E − 01	8.89E − 01	5.99E − 05
45^a^	rs332898	2	T/G	−	Intergenic	7.59E − 01	**4.33E − 02**	1.00E + 00	8.84E − 01	9.01E − 01	9.05E − 01	1.51E − 04
46^a^	rs66461219	2	T/C	LRP1B	Intronic	7.71E − 01	**3.63E − 02**	8.70E − 01	8.99E − 01	9.01E − 01	8.98E − 01	3.16E − 04
47^a^	rs12477286	2	T/G	CIR1	Intronic	9.83E − 01	**4.60E − 02**	5.26E − 01	8.14E − 01	1.00E + 00	9.63E − 01	4.66E − 04
49^a^	rs12475098	2	T/C	SLC4A3	Intergenic	8.49E − 01	**3.21E − 02**	7.82E − 01	7.43E − 01	9.57E − 01	9.07E − 01	2.64E − 04
52^a^	rs11945373	4	A/G	DHX15	Intergenic	1.00E + 00	**3.50E − 03**	1.81E − 01	3.38E − 01	8.99E − 01	NA	1.03E − 05
53^a^	rs13143951	4	A/C	NAA11	ncRNA_intronic	3.87E − 01	**2.02E − 02**	5.59E − 02	5.29E − 02	9.09E − 01	8.41E − 01	1.30E − 04
53^a^	rs9998799	4	T/C	NAA11	ncRNA_intronic	3.37E − 01	2.21E − 02	**3.77E − 02**	5.75E − 02	9.05E − 01	8.07E − 01	1.49E − 04
54^a^	rs17344835	4	T/C	BMPR1B	Intronic	8.82E − 01	**4.12E − 02**	8.03E − 01	9.82E − 01	1.00E + 00	8.80E − 01	1.77E − 04
56^a^	rs72718909	4	T/C	ZFP42	Intergenic	7.35E − 01	**3.61E − 02**	1.00E + 00	9.25E − 01	1.00E + 00	8.42E − 01	1.97E − 04
57	rs10044618	5	T/C	TMEM161B	ncRNA_intronic	9.86E − 01	**2.81E − 04**	7.43E − 01	1.00E + 00	9.91E − 01	1.00E + 00	4.12E − 07
58^a^	rs7715167	5	T/C	FGF18	Intergenic	8.05E − 01	6.48E − 01	4.43E − 01	**3.62E − 02**	9.32E − 01	8.62E − 01	2.02E − 05
59^a^	rs364659	6	T/G	ASCC3	Intergenic	7.75E − 01	**2.77E − 02**	3.97E − 01	7.26E − 01	9.08E − 01	9.78E − 01	1.45E − 04
61	rs17210284	7	T/C	PCLO	Intronic	6.64E − 01	**4.97E − 03**	**3.10E − 03**	5.09E − 02	9.01E − 01	8.65E − 01	4.78E − 06
61	rs17371806	7	A/G	PCLO	Intronic	7.22E − 01	5.62E − 01	1.10E − 01	**1.00E − 02**	9.01E − 01	8.64E − 01	1.30E − 05
62^a^	rs10499973	7	A/G	−	Intergenic	3.53E − 01	**3.59E − 02**	7.80E − 01	2.51E − 01	6.69E − 01	1.00E + 00	3.14E − 04
65^a^	rs13296658	9	A/C	ZDHHC21	Intronic	7.60E − 01	**2.34E − 02**	1.00E + 00	NA	9.01E − 01	8.75E − 01	4.23E − 05
67	rs28481902	9	T/C	TMEM38B	Intergenic	7.62E − 01	8.41E − 01	**4.93E − 02**	1.70E − 01	9.38E − 01	8.65E − 01	7.56E − 05
70^a^	rs34062335	12	T/C	SOX5	Intronic	8.36E − 01	**4.92E − 02**	8.32E − 01	7.78E − 01	9.02E − 01	1.00E + 00	5.04E − 04
73^a^	rs12914677	15	T/G	LOXL1	ncRNA_intronic	8.47E − 01	7.17E − 01	**3.53E − 02**	8.72E − 01	9.97E − 01	8.74E − 01	1.65E − 04
74^a^	rs9928691	16	A/G	−	Intergenic	8.04E − 01	**3.10E − 02**	8.27E − 01	8.67E − 01	1.00E + 00	1.00E + 00	2.08E − 04
75^a^	rs77041900	16	T/C	HAS3	Intronic	8.60E − 01	**3.90E − 02**	7.75E − 01	7.13E − 01	NA	8.67E − 01	2.80E − 04
76^a^	rs11643192	16	A/C	HP	Intergenic	9.96E − 01	**4.13E − 02**	6.00E − 02	5.67E − 01	9.65E − 01	8.64E − 01	3.92E − 04
79^a^	rs11650999	17	A/G	MSI2	Intronic	8.79E − 01	**2.78E − 02**	9.98E − 01	9.15E − 01	1.00E + 00	9.77E − 01	1.35E − 04
80^a^	rs11877758	18	T/G	CELF4	Intronic	4.68E − 01	**3.46E − 02**	1.00E + 00	7.16E − 01	8.31E − 01	1.00E + 00	2.97E − 04
81^a^	rs12965339	18	A/C	DCC	Intronic	5.72E − 01	**3.34E − 02**	7.87E − 01	7.31E − 01	5.91E − 01	9.45E − 01	2.82E − 04
84^a^	rs6130328	20	A/G	PTPRT	Intergenic	7.73E − 01	**3.62E − 02**	4.86E − 01	9.84E − 01	8.99E − 01	8.47E − 01	3.19E − 04
86^a^	rs7530647	1	A/G	TRIT1	Intronic	7.87E − 01	6.73E − 01	3.91E − 01	9.99E − 01	**4.32E − 02**	8.57E − 01	7.16E − 06
87^a^	rs113445115	1	T/C	UBAP2L	Intronic	3.23E − 01	1.00E + 00	**1.32E − 02**	2.02E − 01	9.90E − 01	8.60E − 01	3.55E − 05
89^a^	rs72832204	2	A/G	POU3F3	ncRNA_intronic	9.46E − 01	9.44E − 01	**2.72E − 02**	9.91E − 01	9.05E − 01	1.00E + 00	1.08E − 04
92^a^	rs1356292	3	T/C	ETV5	Intronic	8.40E − 01	3.80E − 01	**9.26E − 03**	1.84E − 01	9.93E − 01	8.64E − 01	2.11E − 05
93^a^	rs13148166	4	T/G	PPA2	Downstream	9.75E − 01	3.84E − 01	**4.23E − 02**	4.34E − 01	1.00E + 00	6.29E − 01	2.21E − 04
95^a^	rs174909	7	T/G	WIPF3	Intergenic	8.47E − 01	8.38E − 01	**3.19E − 02**	4.03E − 01	9.66E − 01	9.87E − 01	1.40E − 04
96^a^	rs4909803	8	T/G	FAM135B	Upstream	8.26E − 01	1.52E − 01	**3.28E − 02**	1.04E − 01	NA	9.89E − 01	1.46E − 04
97^a^	rs1244445	10	T/C	ITIH2	Intergenic	8.65E − 01	9.97E − 01	**3.00E − 02**	9.16E − 01	8.35E − 01	5.29E − 01	4.34E − 05
98^a^	rs10458677	10	A/G	SLC16A9	Intergenic	9.88E − 01	8.21E − 01	**4.19E − 02**	5.28E − 01	1.00E + 00	9.27E − 01	1.80E − 04
101^a^	rs737237	11	T/C	CNTN5	Intronic	5.26E − 01	1.61E − 01	**3.89E − 02**	2.39E − 01	9.67E − 01	8.78E − 01	1.92E − 04
102^a^	rs17711616	11	A/G	ZC3H12C	ncRNA_intronic	7.84E − 01	8.13E − 01	**4.39E − 02**	2.74E − 01	1.00E + 00	1.00E + 00	2.34E − 04
103^a^	rs6581887	12	A/G	LYZ	Intergenic	9.39E − 01	5.47E − 01	**3.41E − 02**	1.00E + 00	9.52E − 01	8.63E − 01	1.44E − 04
105^a^	rs11857990	15	A/G	C15orf40	ncRNA_intronic	9.55E − 01	1.00E + 00	**3.74E − 02**	5.99E − 01	8.51E − 01	9.03E − 01	1.80E − 04
108^a^	rs917415	19	A/C	GNG7	UTR3	9.30E − 01	8.02E − 01	**3.51E − 02**	1.98E − 01	9.00E − 01	NA	1.63E − 04
109^a^	rs892086	19	A/G	SLC44A2	Intronic	8.10E − 01	8.06E − 01	**4.13E − 02**	8.36E − 01	9.99E − 01	9.94E − 01	2.13E − 04
110^a^	rs2024568	20	T/C	CD40	Intergenic	1.47E − 01	6.96E − 01	**3.84E − 02**	6.63E − 02	9.82E − 01	1.91E − 01	1.88E − 04
116^a^	rs73113782	3	A/G	−	Intergenic	8.49E − 01	8.06E − 01	5.72E − 01	**4.85E − 02**	1.00E + 00	9.59E − 01	1.08E − 04
117	rs1280622	3	A/C	PCCB	Intergenic	7.32E − 01	8.58E − 01	8.20E − 01	**3.14E − 03**	7.97E − 01	8.75E − 01	3.24E − 06
118^a^	rs56387490	3	T/C	LPP	Intronic	1.00E + 00	8.37E − 01	3.99E − 01	**4.51E − 02**	1.00E + 00	9.33E − 01	8.28E − 05
123	rs12277680	11	A/G	B3GAT1	Downstream	5.23E − 01	7.93E − 01	1.00E + 00	**2.60E − 02**	8.96E − 01	9.88E − 01	4.57E − 05
127^a^	rs8703	17	T/C	RABEP1	UTR3	1.00E + 00	9.63E − 01	9.52E − 01	**3.43E − 02**	9.84E − 01	9.61E − 01	6.73E − 05

N.B. Conjunctional FDR less than 0.05 are written in bold

^a^Locus novel for irritable bowel syndrome

### Identification of shared genetic loci for IBS and gastrointestinal disorders

**Fig. 6:**
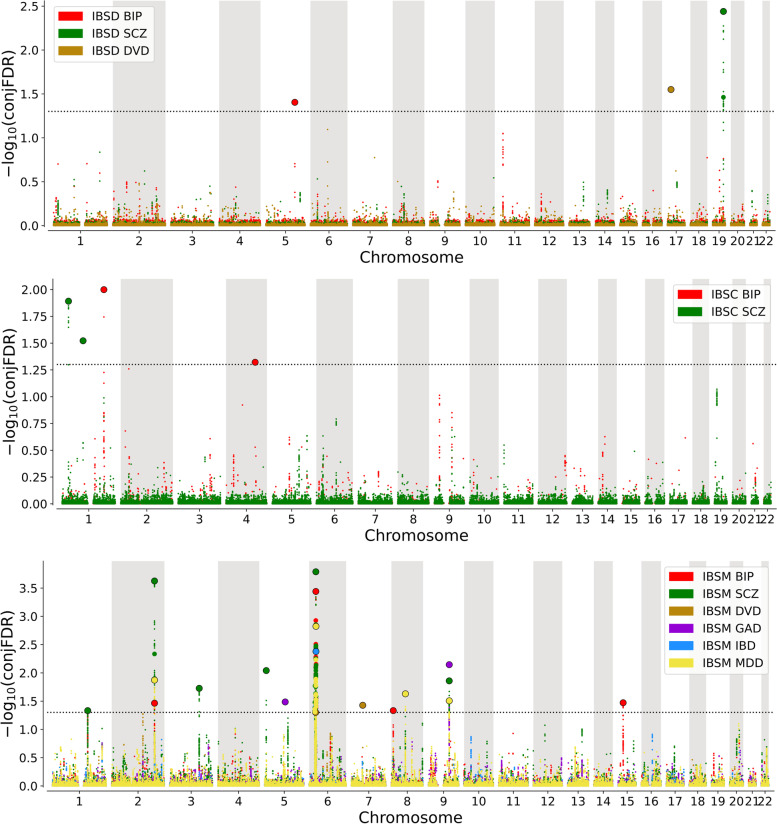
Conjunctional FDR Manhattan plot of –log10 (conjFDR) values for loci shared between different subtypes of irritable bowel syndrome (IBS), and psychiatric disorders and gastrointestinal diseases. Top — IBS with constipation (IBSC), middle — IBS with diarrhea (IBSD), and bottom — IBS with mixed constipation and diarrhea (IBSM). Generalized anxiety disorder (GAD), major depression (MD), bipolar disorder (BIP), schizophrenia (SCZ), diverticular disease (DVD), or inflammatory bowel disease (IBD)

Three loci were shared between IBS and DVD, all with concordant effect directions (Table 2; Fig. 5; Additional file 5: Table S27). The only shared loci identified for IBS and IBD mapped to the MHC region. In conjFDR of the three IBS subtypes, the number of shared loci with psychiatric disorders or gastrointestinal diseases was generally fewer than those shared with the combined IBS sample. BIP and SCZ had shared loci identified with all three subtypes of IBS. However, none of these shared loci were identical across the IBS subtypes. None of the loci shared between IBSD or IBSC and the secondary phenotypes were identified in the conjFDR analyses for the overall IBS sample and the secondary phenotypes. Five of the 11 loci shared between IBSM, and the secondary phenotypes were not identified in the conjFDR analyses of the overall IBS sample (Fig. 6; Additional file 5: Tables S28 – S38).

### Consistency of genetic effects in an independent sample

A total of 175 unique lead SNPs were identified for IBS conditioned on psychiatric and gastrointestinal diseases (Additional file 5: Tables S17 – S22). A total of 30 lead SNPs were excluded (17 due to sample overlap and 13 due to no statistic in the independent dataset) before applying the test of consistency of genetic effects. The remaining 145 SNPs were checked for consistency of their effect direction in the validation GWAS summary statistics from FinnGen. We found a significant *en masse* concordance of effect directions in the discovery and replication samples (100/145, *p* = 2.86E − 06).

### Functional annotations and gene ontology

The majority of loci identified in conjFDR analyses harbored intergenic or intronic SNPs, while two loci, rs5174 [*LRP8*] and rs20551 [*EP300*], were in exonic regions. Several loci shared between IBS and psychiatric disorders rs2265576, rs17210284, rs1909650, rs5174, and rs1356292, had CADD > 12.37 suggesting potentially detrimental effect (Additional file 5: Tables S23 – S26). GO analyses of genes mapped to loci shared between IBS and psychiatric disorders were enriched for biological processes and cellular components relevant to the immune (defense response) and nervous (neurogenesis) systems (Additional file 6: Tables S39 – S40). Genes mapped to loci identified for IBS through condFDR were enriched for biological processes and cellular components related to the immune system (e.g., immune response regulating signaling pathway, response to bacterium, regulation of B cell proliferation, inflammatory response), nervous system (e.g., neurogenesis, chemical synaptic transmission postsynaptic, regulation of neurotransmitter levels), the gastrointestinal tract (e.g., epithelium development, tube development, epithelial tube morphogenesis), and other organ systems (e.g., skeletal system development, bone development, sexual reproduction) (Additional file 6: Tables S41 – S42). Genes mapped to loci shared between IBS and psychiatric disorders were upregulated in various parts of the brain including the anterior cingulate and frontal cortex, basal ganglia, nucleus accumbens, hypothalamus, and amygdala, and downregulated in gastrointestinal organs including the stomach, liver, pancreas, terminal ileum and colon (Additional file 2: Fig. S3).

## Discussion

Our comprehensive characterization of the genetic architecture of IBS demonstrates a high degree of polygenicity, with an estimated 12 k common variants. There was a large degree of polygenic overlap with psychiatric disorders, despite the low genetic correlations of IBS with BIP (0.13) and SCZ (0.17). Our finding that 98% of trait-influencing variants for BIP and 93% for SCZ are overlapping with IBS is not evident from the genetic correlation analyses. Since close to half of the overlapping variants between IBS and BIP (55%) and SCZ (56%) have concordant effect directions and are canceled out by discordant effects at the genome-wide level. Similarly, despite an estimated genetic correlation of zero between IBS and IBD, 77% of the variants influencing IBD are overlapping with IBS, but with nearly half of the variants (52%) having concordant effect directions. However, the difference in the polygenicity of IBS and IBD may have also contributed to the low genetic correlation [21]. In support of this, 100% of the trait influencing variants for DVD are a subset of those for IBS, while the genetic correlations were only moderate despite 99% having concordant effect directions [20]. The relatively high genetic correlations of IBS with MD and GAD may be accounted for by similarity in the polygenic landscape, large genetic overlap, and the concordant effect directions of the large majority (80%) of the variants associated with the pairs of phenotypes. The polygenic architecture of IBS, recognized as a psychosomatic condition, as well as its genetic overlap patterns, are consistent with its inclusion in the psychiatric nosology [42].

Despite increasing GWAS sample sizes, the well-recognized challenge of identifying trait-influencing variants for highly polygenic, complex phenotypes has persisted [51]. The condFDR of IBS on psychiatric disorders brought about a 20-times boost in loci discovery when compared to the primary GWAS of IBS which identified only six genomic loci [11]. CondFDR of IBS on gastrointestinal disorders improved discovery in genomic loci by fourfold. These are consistent with the findings in the conditional QQ plots which demonstrated a more conspicuous SNP enrichment when the secondary phenotypes were psychiatric disorders rather than gastrointestinal diseases [17]. A recent study has identified several shared genomic risk loci between IBS and depressive disorders using whole exome sequencing on a relatively small sample [52]. While such approaches may be interesting, our findings indicate that the majority of the genomic risk loci for IBS are intergenic or intronic, which means that these loci would not be identified with exome sequencing. The condFDR method has been widely used to improve genomic discoveries in other somatic and psychiatric phenotypes [15, 16, 18, 20, 53]. Functional analyses of genes mapped to the much larger number of loci associated with IBS point not only to molecular pathways of the gut-brain axis and epithelium development but also pathways involved in bone and reproductive physiology (Additional file 6: Table 41). Studies have previously reported an increased risk of osteoporosis in individuals affected with IBS [54, 55] and from medications used for IBS [56]. Similarly, changes in gastrointestinal motility and IBS have been linked to sex hormones as well as hormone replacement treatments [57–60]. Functional pathways identified including neurogenesis and neuronal differentiation as well as neuronal and synaptic structures point to the gut-brain axis in IBS and are consistent with the neuroplastic changes reported in the intestine of IBS patients [61]. While changes in mucosal mediators may initiate such neuroplastic changes in the intestine [61], our findings suggest that genetic factors may predispose individuals to these mediators. In regards to synaptic function, dysregulated serotonergic neurotransmission has been suggested to be partly responsible for IBS symptomatology [62]. The identification of novel loci for IBS has shed light on functional pathways other than those relevant to the nervous system. Notably, the functional pathways involving the immune system and gastrointestinal tract development suggest that genetic factors may contribute to IBS through multiple mechanisms. The role of the immune system in IBS is supported by evidence for the development of IBS following gastrointestinal infections at least in some individuals [63]. Taken together, the discovery of a larger number of genomic loci for IBS shed light on the broad range of biological pathways involved in the pathophysiology and hence, the potential for the development of novel treatments.

The significant comorbidity between IBS and psychiatric disorders seen in both clinical [23, 64] and epidemiological data suggest common genetic risk [65, 66]. A total of 70 unique loci were shared between IBS and MD (*n* = 35), BIP (*n* = 27), SCZ (*n* = 15), and GAD (*n* = 7), and three loci with DVD, the majority having a concordant direction of effect. Although there is significant comorbidity between IBS and DVD [26] and IBD [25], fewer loci were identified for IBS by leveraging on these gastrointestinal phenotypes due to the relatively low polygenicity of these phenotypes [17]. The identification of specific shared genomic loci enabled further investigation of the underlying biological mechanisms common to IBS and psychiatric disorders [17]. Genes mapped to shared loci between IBS and both psychiatric and gastrointestinal diseases were enriched for pathways relevant to the nervous and immune systems. Pathways relevant for the gastrointestinal tract were only found for genes mapped to genomic loci identified for IBS. The shared genomic loci identified for IBS subtypes indicate that our main findings for the total IBS sample may reflect the large proportion of IBSM in the overall sample. Interestingly, the shared loci influencing IBSC or IBSD subtypes were not identified in the IBSM subtype or the overall IBS sample highlighting the genetic heterogeneity of the different IBS subtypes. The moderate genetic correlation results between IBSC and IBSD also support genetic differences underlying the variation in the clinical manifestations. These findings may have implications for treatment development and precision medicine in IBS.

The two exonic loci identified for IBS may be relevant for further understanding of shared biological mechanisms between IBS and psychiatric disorders. The SNP rs20551 is a missense variant in *EP300 —* a gene involved in regulation of molecular processes of neuronal plasticity [67] as well as in the differentiation of intestinal epithelial cells [68], and in regulating the expression of intestinal antimicrobial peptides [69]. *LRP8* encodes a receptor protein (ApoER2*)* for ligands containing Reelin (RELN) and apolipoprotein E (Apo-E) [70]. RELN-ApoER2 pathway regulates neurodevelopmental processes in the central [71], and peripheral nervous system [72], and may have a role in the maintenance of the intestinal epithelial barrier [73, 74]. Recent research has demonstrated the critical role of nociceptor neuronal signaling in the protection of intestinal mucosa at homeostasis and during inflammatory pathologies [75]. Furthermore, the deranged intestinal barrier and translocation of bacterial products have been linked to changes in the blood–brain barrier with consequent behavioral and cognitive changes [76]. Similar gut-brain-axis mechanisms may also play a role in IBS and partly explain the high comorbidity with psychiatric disorders. On the other hand, pleiotropic genes such as *EP300* and *LRP8* influencing both the intestinal and brain functions may underly the high comorbidity between common psychiatric and gastrointestinal phenotypes. Further research is needed to fully understand the extent these mechanisms contribute and whether they vary in the different clinical subtypes of IBS.

One limitation of this undertaking is that GWAS data used for our analyses are from individuals of European ancestry thereby limiting the generalization of our findings to other ancestries. Although we used BIP GWAS data removing the UK biobank, there are still other cohorts included in the meta-analysis that could have overlap, particularly of controls. It is unlikely that there is any overlap between samples included in IBS and the other phenotypes as indicated by the genetic covariance parameters (Additional file 4: Table S43). Another potential limitation arising from the IBS GWAS could be that comorbid psychiatric disorders may not have been adequately excluded, which may explain some of the genetic overlap, especially with MD or GAD. However, it is unlikely that comorbidities with BIP and SCZ explain the genetic overlap with IBS as both are relatively rare conditions [77]. Similarly, comorbidity is unlikely to explain the genetic overlap between IBS and gastrointestinal diseases since they were excluded from the IBS sample [11]. Sex-stratified analyses were not performed because sex-specific GWAS summary statistics of IBS are not yet available. Despite these limitations, our analytical methods have substantially improved the discovery of genetic loci for IBS and revealed much broader biological pathways involving not only the gut-brain axis and immune system but also intestinal development, and bone and reproductive physiology. These findings suggest a wide range of biological pathways involved in IBS which can potentially be leveraged to develop biological treatments targeting these pathways. Our findings of genetic heterogeneity of the clinical subtypes of IBS call for further research into subtype-specific biological pathways to help advance precision medicine.

## Conclusions

Our findings of the polygenic architecture of IBS and the extensive genetic overlap between IBS and both psychiatric disorders and gastrointestinal diseases provide novel insight into the shared genetic architecture beyond genetic correlations. This genetic overlap enabled the identification of 132 genomic risk loci for IBS, of which 116 are novel. Functional pathway analyses suggest that genetic factors may influence a wide range of biological pathways including the gut-brain axis and local gastrointestinal mechanisms in the etiopathology of IBS. Shared genomic loci associated with IBS and psychiatric disorders show enrichment of genes for neurogenesis and defense response suggesting dysregulation of molecular pathways of the gut-brain axis and the immune system. Furthermore, the pattern of genetic correlations and shared genomic loci with psychiatric disorders support the underlying genetic heterogeneity of IBS subtypes. These genetic discoveries provide a better understanding of the pathophysiology of IBS potentially forming the basis for the development of more effective interventions.

## Supplementary Information

**Additional file 1.** Detailed description on univariate and bivariate causal mixture models, and conditional and conjunctional false discovery rate analysis methods.

**Additional file 2:** **Fig. S1. **Conditional Q-Q plots of nominal -log10 p-values vs empirical -log10 p-values in generalized anxiety disorder, major depression, bipolar disorder, schizophrenia, diverticular disease, or inflammatory bowel disease below the standard genome-wide association study threshold of p < 5.0 × 10^−8^ as a function of significance of association with irritable bowel syndrome. **Fig. S2.** Conditional FDR Manhattan plot of –log10 values of loci identified for irritable bowel syndrome by conditioning on generalized anxiety disorder, major depression, bipolar disorder, schizophrenia, diverticular disease, and inflammatory bowel disease. **Fig. S3.** Tissue enrichment for differential gene expression (DEG) in 54 GTEx tissue types of genes mapped to shared genomic loci associated with irritable bowel syndrome and psychiatric disorders.

**Additional file 3:** **Tables S1 – S15. **Results from univariate and bivariate MiXeR analyses for different psychiatric and somatic phenotypes.

**Additional file 4:** **Table S16.** Genetic correlations from LD score regression analyses for subtypes of irritable bowel syndrome (IBS), and psychiatric and gastrointestinal diseases. **Table S43.** Genetic covariance parameters between irritable syndrome and psychiatric and gastrointestinal diseases computed from summary statistics used for conditional FDR analyses.

**Additional file 5:** **Tables S17 – S38.** Genomic risk loci for irritable bowel syndrome identified on conditional FDR and shared genomic loci with various phenotypes identified in conjunctional FDR analyses.

**Additional file 6:** **Table S39 - S42.** Gene Ontology: Enrichment of biological processes and cellular components for genes annotated to genomic risk loci identified for irritable bowel syndrome, and shared genomic loci between irritable bowel syndrome and psychiatric disorders.

## Data Availability

The GWAS of generalized anxiety disorder can be accessed at https://www.ncbi.nlm.nih.gov/gap/, dbGaP Study Accession phs001672 [29], and the GWAS summary statistics for the 23andMe major depression dataset are available through 23andMe to qualified researchers under an agreement with 23andMe that protects the privacy of the 23andMe participants [32]. Interested investigators should email dataset-request@23andme.com and reference this paper for more information. The codes used to perform our analyses are available online https://github.com/precimed/pleiofdr [15], and https://github.com/precimed/mixer [21].

## Abbreviations

AIC: Akaike information criterion
BIP: Bipolar disorder
CADD: Combined Annotation Dependent Depletion
CondFDR: Conditional FDR
ConjFDR: Conjunctional FDR
DVD: Diverticular disease
FDR: False discovery rate
FUMA: Functional Mapping and Annotations
GAD: Generalized anxiety disorder
GO: Gene ontology
GWAS: Genome-wide association study
IBD: Inflammatory bowel disease
IBS: Irritable bowel syndrome
LD: Linkage disequilibrium
MD: Major depression
QQ plot: Quantile-quantile plot
SCZ: Schizophrenia
SNP: Single nucleotide polymorphism
