# Supplementary material for: Shared genetic architecture between irritable bowel syndrome and psychiatric disorders reveals molecular pathways of the gut-brain axis
Source: Genome Med. 2023 Aug 1;15:60. doi: 10.1186/s13073-023-01212-4 (PMC10391890; doi:10.1186/s13073-023-01212-4)
Supplement: Supplementary file 1 — Additional file 1. Detailed description on univariate and bivariate causal mixture models, and conditional and conjunctional false discovery rate analysis methods. [file 13073_2023_1212_MOESM1_ESM.docx]

**Supplementary methods for:**

**Shared genetic architecture between irritable bowel syndrome and psychiatric disorders reveals molecular pathways of the gut-brain axis**

**Authors**

Markos Tesfaye^1,2*^, Piotr Jaholkowski^1^, Guy F. L. Hindley^1,3^, Alexey A. Shadrin^1,4^, Zillur Rahman^1^, Shahram Bahrami^1^, Aihua Lin^1^, Børge Holen^1^, Nadine Parker^1^, Weiqiu Cheng^1^, Linn Rødevand^1^, Oleksandr Frei^1,5^, Srdjan Djurovic^2,6^ Anders M. Dale^7,8,9,10^, Olav B. Smeland^1^, Kevin S. O’Connell,^1^ Ole A. Andreassen^1,4 *^

**Affiliations**

^1^ NORMENT, Centre for Mental Disorders Research, Division of Mental Health and Addiction, Oslo University Hospital, and Institute of Clinical Medicine, University of Oslo, Oslo, Norway

^2^ NORMENT, Department of clinical sciences, University of Bergen, Bergen, Norway

^3^ Institute of Psychiatry, Psychology and Neuroscience, King’s College London, London, UK

^4^ KG Jebsen Centre for Neurodevelopmental Disorders, University of Oslo and Oslo University Hospital, Oslo, Norway

^5^ Center for Bioinformatics, Department of Informatics, University of Oslo, Oslo, Norway

^6^ Department of Medical Genetics, Oslo University Hospital, Oslo, Norway

^7^ Department of Radiology, University of California, San Diego, La Jolla, CA, USA

^8^ Multimodal Imaging Laboratory, University of California San Diego, La Jolla, CA, USA

^9^ Department of Psychiatry, University of California, San Diego, La Jolla, CA, USA

^10^ Department of Neurosciences, University of California San Diego, La Jolla, CA, USA

*** Corresponding authors**

Markos Tesfaye, M.D., Ph.D. ([m.t.woldeyohannes@medisin.uio.no](mailto:m.t.woldeyohannes@medisin.uio.no)) and

Ole Andreassen, M.D., Ph.D. ([o.a.andreassen@medisin.uio.no](mailto:o.a.andreassen@medisin.uio.no))

Division of Mental Health and Addiction, Oslo University Hospital &

Institute of Clinical Medicine, University of Oslo

Building 49, Oslo University Hospital, Ullevål,

Kirkeveien 166, PO Box 4956 Nydalen, 0424 Oslo, Norway

**Statistical Analyses**

**MiXeR Analyses**

*Univariate MiXeR*

For each SNP, $i$, MiXeR models its additive genetic effect of allele substitution,$\beta_{I}$, as a point-normal mixture, $\beta_{i}=\left( 1-\pi_{1} \right)N\left( 0,0 \right)+\pi_{1}N\left( 0, \sigma_{\beta}^{2} \right)$, where $\pi_{1}$ represents the proportion of non-null SNPs (i.e., polygenicity) and $\sigma_{\beta}^{2}$ represents variance of effect sizes of non-null SNPs (i.e., discoverability). Then, for each SNP, $j$, MiXeR incorporates LD information and allele frequencies for input SNPs calculated based on 1000 Genomes Phase3 reference panel [1] to estimate the expected probability distribution of the signed test statistic, $z_{j}=\delta_{j}+\epsilon_{j}=\sqrt{N}\sum_{i} \sqrt{H_{i}}r_{\mathrm{ij}}\beta_{i}+\epsilon_{j}$, where $N$ is sample size, $H_{I}$ indicates heterozygosity of i-th SNP, $r_{\mathrm{ij}}$ indicates allelic correlation between i-th and j-th SNPs, and $\epsilon_{j}\sim N\left( 0, \sigma_{0}^{2} \right)$ is the residual variance. Further, the three parameters, $\pi_{1}, \sigma_{\beta}^{2}, \sigma_{0}^{2}$, are fitted by direct maximization of the likelihood function. The number of trait-influencing variants is estimated as $M\pi_{1}$, where M is the number of SNPs in the reference panel. The phenotypic variance explained on average by a trait-influencing variant is calculated as $\bar{H}\sigma_{\beta}^{2},$ where $\bar{H}=\frac{1}{M}\sum_{i} H_{i}=0.2075$ is the average heterozygosity across SNPs in the reference panel. Under the assumptions of the MiXeR model, SNP-heritability is then calculated as $h_{\mathrm{SNP}}^{2}=M\pi_{1}\times\bar{H}\sigma_{\beta}^{2}$ [2].

*Bivariate MiXeR*

MiXeR models additive genetic effects as a mixture of four components, representing null SNPs in both traits ($\pi_{0})$; SNPs with a specific effect on the first and on the second trait ($\pi_{1}$ and $\pi_{2}$, respectively); and SNPs with non-zero effect on both traits ($\pi_{12}$). In the last component, MiXeR models variance-covariance matrix as $\boldsymbol{\Sigma}_{\mathbf{12}}=\left[ \begin{matrix} \sigma_{1}^{2} & {\rho_{12}\sigma}_{1}\sigma_{2} \\ {\rho_{12}\sigma}_{1}\sigma_{2} & \sigma_{2}^{2} \end{matrix} \right]$ where $\rho_{12}$ indicates correlation of effect sizes within the shared component, and $\sigma_{1}^{2}$ and $\sigma_{2}^{2}$ correspond to the discoverability parameters estimated in the univariate analyses of the two traits. After fitting parameters of the model genetic correlation is calculated as $r_{g}=\frac{\rho_{12}\pi_{12}}{\sqrt{\left( \pi_{1}+\pi_{12} \right)\left( \pi_{2}+\pi_{12} \right)}}.$

The dice coefficient (DC) was calculated using the formula DC = $\frac{2\pi_{12}}{{\pi_{1}+\pi_{2}+2\pi}_{12}}$.

MiXeR performed 20 iterations of each univariate and bivariate analysis with 2 million randomly selected SNPs with a MAF threshold of 5% followed by random pruning at an LD threshold of r^2^=0.8, resulting in a sample of ~600K input SNPs per iteration. Those input SNPs were used to construct the distribution of genetic effects used to calculate the observed signed test statistics. The mean and standard deviation estimates for each parameter were calculated from the sample of 20 iterations [2].

To identify analyses using insufficiently powered GWAS summary statistics, MiXeR computes the Akaike information criterion ($AIC=2k-2\ln\left( L \right)$), where $k$ is the number of free parameters in the model and $L$ is the value of the likelihood function. In univariate analysis, the ΔAIC represents the difference between AIC of the model fitted using MiXeR and AIC of the infinitesimal model, which assumes that all variants are non-null resulting in the constrained univariate model with 2 free parameters ($\sigma_{\beta}^{2}, \sigma_{0}^{2}$) while fixing polygenicity parameter equal to 1. A positive ΔAIC value indicates the input GWAS dataset provides sufficient power to discriminate the model fitted using MiXeR from the infinitesimal model.

In bivariate analysis, we calculated the difference between $\mathrm{AIC}$ between the full bivariate model, $k=3$, and the reduced bivariate model, $k=2$, due to $\pi_{12}$ being constrained to the smallest or largest possible value ( $\pi_{12}^{\min}=r_{g}\sqrt{\pi_{1}^{u} \pi_{2}^{u}}$ and $\pi_{12}^{\max}=\min\left( \pi_{1}^{u}, \pi_{2}^{u} \right)$), respectively. Each bivariate analysis returns two ΔAIC values, including ΔAIC_best_vs_min_ (which compares MiXeR modelled fit with a constrained model with minimal polygenic overlap) and ΔAIC_best_vs_max_ (which compares MiXeR modelled fit with a constrained model with maximum polygenic overlap). When both ΔAIC_best_vs_min_ and ΔAIC_best_vs_max_ are positive, which is observed for IBS and IBD (Supplementary file 1), it implies that the GWAS summary statistics have enough information to distinguish the modelled polygenic overlap, as shown on the MiXeR Venn diagrams, versus the constrained models with minimal ($\pi_{12}^{\min}$) and maximum ($\pi_{12}^{\max}$) polygenic overlap. A positive ΔAIC_best_vs_min_ with a negative ΔAIC_best_vs_max_, which were observed for IBS and BIP or IBS and SCZ (Supplementary Table 1), imply genetic overlap beyond the minimal polygenic overlap. This suggests further investigations using more powerful GWAS datasets will help finalize the estimates of polygenic overlap [2].

**Conditional and Conjunctional False Discovery Rates (CondFDR/ConjFDR)**

Conditional Q-Q plots

Q-Q plots of nominal p-values from GWAS summary statistics are one of the common approaches through which one can visualize the enrichment of statistical association relative to that expected under the global null hypothesis (none of genetic variants influences the phenotype). The Q-Q curve has the nominal p-values, denoted by “p”, on the y-coordinate and the corresponding value of the empirical cumulative distribution function (cdf), denoted by “q”, on the x-coordinate. The -log10(p) is commonly plotted against the -log10(q) to accentuate tail probabilities of the theoretical and empirical distributions. Under the global null hypothesis, the theoretical distribution of p-values is uniform on the interval [0,1]. In the presence of all null relationships, nominal –log10(p) form a straight (diagonal) line on a Q-Q plot when plotted against the empirical distribution of –log10(q). Leftward deflections of the observed distribution from the projected null line reflect increased tail probabilities in the distribution of test statistics and consequently an over-abundance of low p-values compared to that expected by chance, termed as ‘enrichment’.

Conditional Q-Q plots are constructed by creating subsets of SNPs based on levels of an auxiliary measure for each SNP, and computing Q-Q plots separately for each cut-off of the auxiliary measure [3]. In our analysis significance of association with the secondary (conditional) phenotype is used as an auxiliary measure. If SNP enrichment is obtained as a result of variation in the auxiliary measure, this is expressed as successive leftward deflections in a conditional Q-Q plot as levels of the auxiliary measure increase. The enrichment can be directly interpreted in terms of the true discovery rate (1−FDR) [4]. Cross-trait enrichment exists if the proportion of SNPs associated with a phenotype increases as a function of the strength of the association with a secondary phenotype.

We constructed conditional Q-Q plots of empirical quantiles of nominal -log10 p-values for SNP association for all SNPs, and for subsets (strata) of SNPs determined by the nominal p-values of their association with the conditional phenotypes, and vice versa. Specifically, we computed the empirical cumulative distribution of nominal p-values for IBS for all SNPs and for SNPs with significance levels below the indicated cut-offs for the conditional phenotypes (e.g., GAD) at p < 0.1, p < 0.01, p < 0.001. The nominal p-values (– log10(p)) are plotted on the y-axis, and the empirical cumulative distribution function of nominal p-values (–log10(q), where q=ecdf(p)) is plotted on the x-axis.

Conditional and Conjunctional FDR

CondFDR is an extension of the standard FDR, which incorporates information from GWAS summary statistics of a second (conditional) phenotype to adjust significance levels in the first (primary) phenotype. The condFDR is defined as the probability that a SNP is null in the first phenotype given that the p-values in the first and second phenotypes are as small as or smaller than the observed ones. The condFDR estimates are obtained for each nominal SNP p-value in the primary phenotype after computing the stratified empirical cdfs of the nominal p-values [5, 6]. The separate strata are determined by the relative enrichment of SNP associations as a function of increased nominal SNP p-values in a secondary phenotype. The standard FDR framework derives from a model that assumes that the distribution of test statistics in a GWAS can be formulated as a mixture of null and non-null effects, with true associations having more extreme test statistics than false associations on average. Ranking SNPs by the standard FDR or by p-values gives the same ordering of SNPs. Given the cross-trait SNP enrichment between the primary and secondary phenotypes, the condFDR procedure re-orders SNPs and results in a different ranking than that using p-values alone.

ConjFDR analysis aims to boost the statistical power for the discovery of shared genetic variants between two phenotypes. It is defined as the posterior probability that a SNP is null for either phenotype or both simultaneously, given that its p-values for association with both phenotypes are as small as or smaller than the observed p-values [7, 8]. We implement conjFDR analysis as two consequent condFDR analyses. First, IBS is the primary phenotype and one of the others (e.g., GAD) is the conditional (secondary) phenotype. Second, an inverse condFDR is performed where IBS becomes the conditional phenotype and the other phenotype (e.g., GAD) becomes the primary phenotype. Then a conservative estimate of the conjFDR values for each SNP can be derived as the maximum of two condFDR values [3]. The significance threshold for the conjFDR analysis was defined as a conjFDR value < 0.05. More methodological details of condFDR and conjFDR are available in the original publications and a more recent review [3, 7].

**References**

1. Sudmant PH, Rausch T, Gardner EJ, Handsaker RE, Abyzov A, Huddleston J, et al. An integrated map of structural variation in 2,504 human genomes. Nature. 2015;526(7571):75-81.

2. Frei O, Holland D, Smeland OB, Shadrin AA, Fan CC, Maeland S, et al. Bivariate causal mixture model quantifies polygenic overlap between complex traits beyond genetic correlation. Nat Commun. 2019;10(1):2417.

3. Smeland OB, Frei O, Shadrin A, O'Connell K, Fan CC, Bahrami S, et al. Discovery of shared genomic loci using the conditional false discovery rate approach. Hum Genet. 2020;139(1):85-94.

4. Efron B. Large-Scale Inference: Empirical Bayes Methods for Estimation, Testing, and Prediction. . Cambridge: Cambridge University Press; 2010.

5. Sun L, Craiu RV, Paterson AD, Bull SB. Stratified false discovery control for large-scale hypothesis testing with application to genome-wide association studies. Genet Epidemiol. 2006;30(6):519-30.

6. Yoo YJ, Pinnaduwage D, Waggott D, Bull SB, Sun L. Genome-wide association analyses of North American Rheumatoid Arthritis Consortium and Framingham Heart Study data utilizing genome-wide linkage results. BMC Proc. 2009;3 Suppl 7(Suppl 7):S103.

7. Andreassen OA, Djurovic S, Thompson WK, Schork AJ, Kendler KS, O'Donovan MC, et al. Improved detection of common variants associated with schizophrenia by leveraging pleiotropy with cardiovascular-disease risk factors. Am J Hum Genet. 2013;92(2):197-209.

8. Andreassen OA, Thompson WK, Dale AM. Boosting the power of schizophrenia genetics by leveraging new statistical tools. Schizophr Bull. 2014;40(1):13-7.
