# Supplementary material for: Shared genetic architecture between irritable bowel syndrome and psychiatric disorders reveals molecular pathways of the gut-brain axis
Source: Genome Med. 2023 Aug 1;15:60. doi: 10.1186/s13073-023-01212-4 (PMC10391890; doi:10.1186/s13073-023-01212-4)
Supplement: Supplementary file 2 — Additional file 2: Fig. S1. Conditional Q-Q plots of nominal -log10 p-values vs empirical -log10 p-values in generalized anxiety disorder, major depression, bipolar disorder, schizophrenia, diverticular disease, or inflammatory bowel disease below the standard genome-wide association study threshold of p < 5.0 × 10^−8^ as a function of significance of association with irritable bowel syndrome. Fig. S2. Conditional FDR Manhattan plot of –log10 values of loci identified for irritable bowel syndrome by conditioning on generalized anxiety disorder, major depression, bipolar disorder, schizophrenia, diverticular disease, and inflammatory bowel disease. Fig. S3. Tissue enrichment for differential gene expression (DEG) in 54 GTEx tissue types of genes mapped to shared genomic loci associated with irritable bowel syndrome and psychiatric disorders. [file 13073_2023_1212_MOESM2_ESM.docx]

**Supplementary figures for:**

**Shared genetic architecture between irritable bowel syndrome and psychiatric disorders reveals molecular pathways of the gut-brain axis**

**Authors**

Markos Tesfaye^1,2*^, Piotr Jaholkowski^1^, Guy F. L. Hindley^1,3^, Alexey A. Shadrin^1,4^, Zillur Rahman^1^, Shahram Bahrami^1^, Aihua Lin^1^, Børge Holen^1^, Nadine Parker^1^, Weiqiu Cheng^1^, Linn Rødevand^1^, Oleksandr Frei^1,5^, Srdjan Djurovic^2,6^ Anders M. Dale^7,8,9,10^, Olav B. Smeland^1^, Kevin S. O’Connell,^1^ Ole A. Andreassen^1,4 *^

**Affiliations**

^1^ NORMENT, Centre for Mental Disorders Research, Division of Mental Health and Addiction, Oslo University Hospital, and Institute of Clinical Medicine, University of Oslo, Oslo, Norway

^2^ NORMENT, Department of clinical sciences, University of Bergen, Bergen, Norway

^3^ Institute of Psychiatry, Psychology and Neuroscience, King’s College London, London, UK

^4^ KG Jebsen Centre for Neurodevelopmental Disorders, University of Oslo and Oslo University Hospital, Oslo, Norway

^5^ Center for Bioinformatics, Department of Informatics, University of Oslo, Oslo, Norway

^6^ Department of Medical Genetics, Oslo University Hospital, Oslo, Norway

^7^ Department of Radiology, University of California, San Diego, La Jolla, CA, USA

^8^ Multimodal Imaging Laboratory, University of California San Diego, La Jolla, CA, USA

^9^ Department of Psychiatry, University of California, San Diego, La Jolla, CA, USA

^10^ Department of Neurosciences, University of California San Diego, La Jolla, CA, USA

*** Corresponding authors**

Markos Tesfaye, M.D., Ph.D. ([m.t.woldeyohannes@medisin.uio.no](mailto:m.t.woldeyohannes@medisin.uio.no)) and

Ole Andreassen, M.D., Ph.D. ([o.a.andreassen@medisin.uio.no](mailto:o.a.andreassen@medisin.uio.no))

Division of Mental Health and Addiction, Oslo University Hospital &

Institute of Clinical Medicine, University of Oslo

Building 49, Oslo University Hospital, Ullevål,

Kirkeveien 166, PO Box 4956 Nydalen, 0424 Oslo, Norway


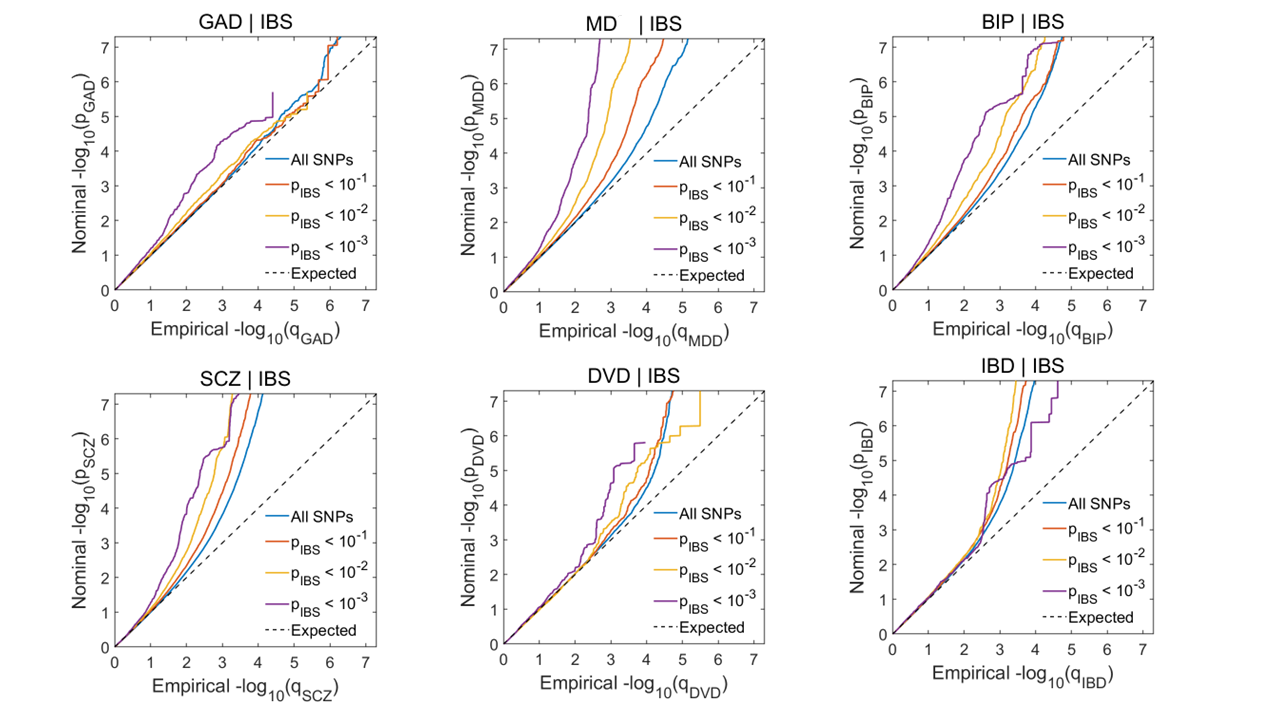


**Figure S1.** Conditional Q-Q plots of nominal -log10 p-values vs empirical -log10 p-values in generalized anxiety disorder (GAD), major depression (MD), bipolar disorder (BIP), schizophrenia (SCZ), diverticular disease (DVD) or inflammatory bowel disease (IBD) below the standard genome-wide association study threshold of p < 5.0 × 10^−8^ as a function of significance of association with irritable bowel syndrome (IBS) below the level of –log10 p-values of 1, 2, or 3, corresponding to p < 0.10, p < 0.01 and p < 0.001, respectively. The blue line includes all SNPs and dashed lines indicate the null hypothesis.


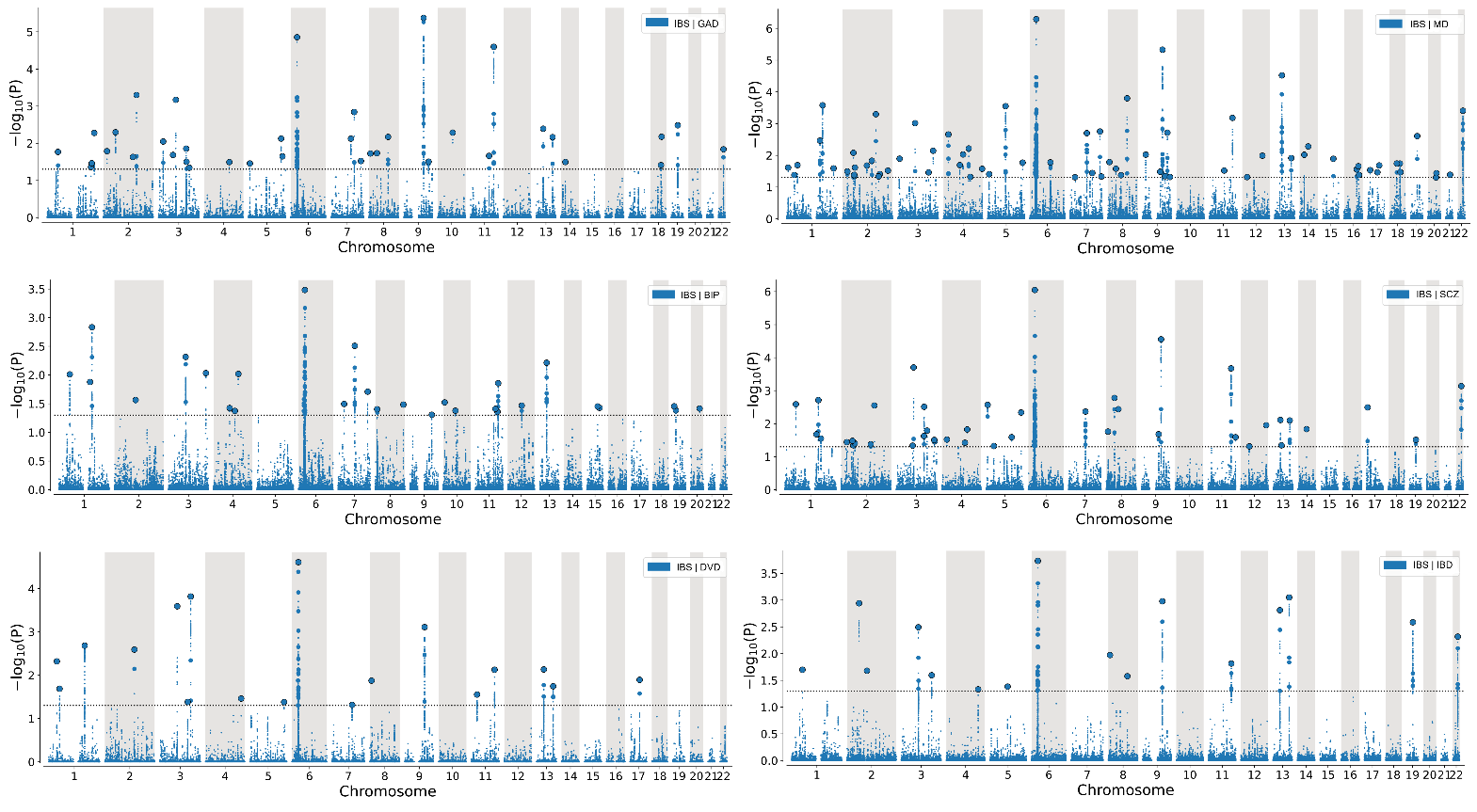


**Figure S2.** Conditional FDR Manhattan plot of –log10 (condFDR) values of loci identified for irritable bowel syndrome (IBS) by conditioning on generalized anxiety disorder (GAD), major depression (MD), bipolar disorder (BIP), schizophrenia (SCZ), diverticular disease (DVD) and inflammatory bowel disease (IBD) SNPs with conditional –log10(condFDR) > 1.3 (i.e., condFDR < 0.05) are shown with large points.


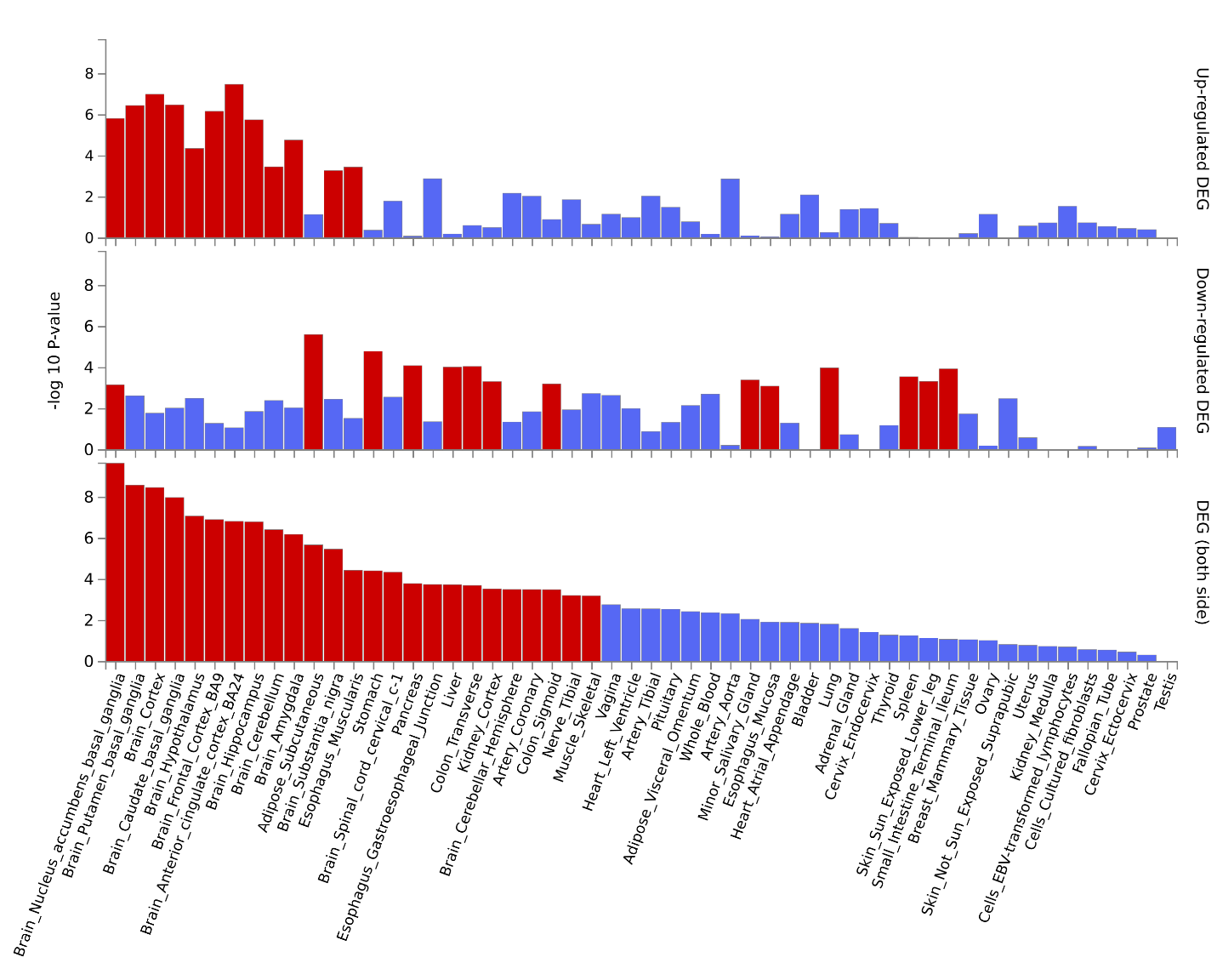


**Figure S3.** Tissue enrichment for differential gene expression (DEG) in 54 GTEx tissue types of genes mapped to shared genomic loci associated with irritable bowel syndrome and psychiatric disorders (generalized anxiety disorder, major depression, bipolar disorder and schizophrenia) with conjunctional false discovery rate less than 0.05 (Additional file 5: Tables S23 - S26). Analysis was performed using FUMA.[1]

**References**

1. Watanabe K, Taskesen E, van Bochoven A, Posthuma D. Functional mapping and annotation of genetic associations with FUMA. Nat Commun. 2017;8(1):1826.
