# Supplementary material for: Shared genetic architecture between irritable bowel syndrome and psychiatric disorders reveals molecular pathways of the gut-brain axis
Source: Genome Med. 2023 Aug 1;15:60. doi: 10.1186/s13073-023-01212-4 (PMC10391890; doi:10.1186/s13073-023-01212-4)
Supplement: Supplementary file 4 — Additional file 4: Table S16. Genetic correlations from LD score regression analyses for subtypes of irritable bowel syndrome (IBS), and psychiatric and gastrointestinal diseases. Table S43. Genetic covariance parameters between irritable syndrome and psychiatric and gastrointestinal diseases computed from summary statistics used for conditional FDR analyses. [file 13073_2023_1212_MOESM4_ESM.docx]

**Supplementary tables for:**

**Shared genetic architecture between irritable bowel syndrome and psychiatric disorders reveals molecular pathways of the gut-brain axis**

**Authors**

Markos Tesfaye^1,2*^, Piotr Jaholkowski^1^, Guy F. L. Hindley^1,3^, Alexey A. Shadrin^1,4^, Zillur Rahman^1^, Shahram Bahrami^1^, Aihua Lin^1^, Børge Holen^1^, Nadine Parker^1^, Weiqiu Cheng^1^, Linn Rødevand^1^, Oleksandr Frei^1,5^, Srdjan Djurovic^2,6^ Anders M. Dale^7,8,9,10^, Olav B. Smeland^1^, Kevin S. O’Connell,^1^ Ole A. Andreassen^1,4 *^

**Affiliations**

^1^ NORMENT, Centre for Mental Disorders Research, Division of Mental Health and Addiction, Oslo University Hospital, and Institute of Clinical Medicine, University of Oslo, Oslo, Norway

^2^ NORMENT, Department of clinical sciences, University of Bergen, Bergen, Norway

^3^ Institute of Psychiatry, Psychology and Neuroscience, King’s College London, London, UK

^4^ KG Jebsen Centre for Neurodevelopmental Disorders, University of Oslo and Oslo University Hospital, Oslo, Norway

^5^ Center for Bioinformatics, Department of Informatics, University of Oslo, Oslo, Norway

^6^ Department of Medical Genetics, Oslo University Hospital, Oslo, Norway

^7^ Department of Radiology, University of California, San Diego, La Jolla, CA, USA

^8^ Multimodal Imaging Laboratory, University of California San Diego, La Jolla, CA, USA

^9^ Department of Psychiatry, University of California, San Diego, La Jolla, CA, USA

^10^ Department of Neurosciences, University of California San Diego, La Jolla, CA, USA

*** Corresponding authors**

Markos Tesfaye, M.D., Ph.D. ([m.t.woldeyohannes@medisin.uio.no](mailto:m.t.woldeyohannes@medisin.uio.no)) and

Ole Andreassen, M.D., Ph.D. ([o.a.andreassen@medisin.uio.no](mailto:o.a.andreassen@medisin.uio.no))

Division of Mental Health and Addiction, Oslo University Hospital &

Institute of Clinical Medicine, University of Oslo

Building 49, Oslo University Hospital, Ullevål,

Kirkeveien 166, PO Box 4956 Nydalen, 0424 Oslo, Norway

| **Table S16.** Genetic correlations from LD score regression analyses for subtypes of irritable bowel syndrome (IBS), and psychiatric and gastrointestinal diseases. | | | | | | | | | |
| --- | --- | --- | --- | --- | --- | --- | --- | --- | --- |
|  | **IBSC** | | | **IBSD** | | | **IBSM** | | |
|  | **R_g_** | **S.E.** | **P-value** | **R_g_** | **S.E.** | **P-value** | **R_g_** | **S.E.** | **P-value** |
| SCZ | 0.14 | 0.06 | 2.00E-02 | 0.15 | 0.05 | 1.30E-03 | 0.15 | 0.04 | 2.00E-04 |
| BIP | 0.11 | 0.07 | 1.27E-01 | 0.13 | 0.05 | 1.34E-02 | 0.06 | 0.06 | 1.83E-01 |
| MD | 0.35 | 0.08 | 7.12E-06 | 0.39 | 0.06 | 2.69E-11 | 0.46 | 0.05 | 1.49E-18 |
| GAD | 0.53 | 0.12 | 1.13E-05 | 0.43 | 0.08 | 9.16E-09 | 0.56 | 0.06 | 1.13E-17 |
| IBD | -0.01 | 0.03 | 7.10E-01 | -0.01 | 0.02 | 5.17E-01 | -0.02 | 0.02 | 2.27E-01 |
| DVD | 0.11 | 0.08 | 1.61E-01 | 0.25 | 0.07 | 2.00E-04 | 0.48 | 0.05 | 9.39E-22 |
| IBSM | 0.87 | 0.18 | 1.85E-06 | 0.88 | 0.1 | 1.25E-18 | 1 | 0 | 0.00E+00 |
| IBSD | 0.39 | 0.15 | 9.60E-03 | 1 | 0 | 0.00E+00 | 0.88 | 0.1 | 1.25E-18 |
| IBSC | 1 | 0 | 0.00E+00 | 0.39 | 0.15 | 9.60E-03 | 0.87 | 0.18 | 1.85E-06 |
| SCZ | 0.14 | 0.06 | 2.00E-02 | 0.15 | 0.05 | 1.30E-03 | 0.15 | 0.04 | 2.00E-04 |
| IBSC - IBS with constipation, IBSD - IBS with diarrhea, IBSM - IBS with mixed constipation and diarrhea, GAD - generalized anxiety disorder, MD - major depression, BIP - bipolar disorder, SCZ - schizophrenia, DVD - diverticular disease, IBD - inflammatory bowel disease, R_g_ – genetic correlation, S.E. – standard error | | | | | | | | | |

| **Table S43:** Genetic covariance parameters between irritable syndrome (IBS) and psychiatric and gastrointestinal diseases computed from summary statistics used for conditional false discovery rate analyses. | | | |
| --- | --- | --- | --- |
| **Phenotypes compared with IBS** | **Genetic covariance (standard error)** | **Intercept (Standard error)** | **P-value for intercept** |
| Generalized Anxiety Disorder | 0.0386 (0.0036) | 0.006 (0.0057) | 0.146 |
| Major Depressive Disorder | 0.0323 (0.0023) | 0.0025 (0.0056) | 0.328 |
| Bipolar Disorder | 0.0173 (0.0042) | 0.0194 (0.0059) | 0.001 |
| Schizophrenia | 0.0269 (0.0044) | -4.56e-06 (0.0059) | 0.500 |
| Diverticular Disease | 0.0344 (0.0041) | 0.0038(0.0048) | 0.214 |
| Inflammatory Bowel Disease | -0.0013 (0.0017) | 0.007 (0.0033) | 0.017 |
